# Supplementary material for: Defect-tolerant electron and defect-sensitive phonon transport in quasi-2D conjugated coordination polymers
Source: Nat Commun. 2025 Jul 18;16:6628. doi: 10.1038/s41467-025-61920-w (PMC12274412; doi:10.1038/s41467-025-61920-w)
Supplement: Supplementary file 1 — Supplementary Information [file 41467_2025_61920_MOESM1_ESM.pdf]

Supplementary Information

Defect-tolerant electron and defect-sensitive phonon transport in quasi-2D conjugated coordination polymers

Hio-Ieng Un*, Kamil Iwanowski, Jordi Ferrer Orri, Ian E. Jacobs, Naoya Fukui, David Cornil, David Beljonne, Michele Simoncelli*, Hiroshi Nishihara, Henning Sirringhaus*

Corresponding author: hiu20@cam.ac.uk, michele.simoncelli@columbia.edu, hs220@cam.ac.uk

I. Supplementary Methods
1. Materials and device methods
   1.1. Materials
   1.2. Device methods

II. Supplementary Discussion
2. Chemical and structural characterizations and analysis
   2.1. Scanning electron microscopy (SEM) and Energy dispersive X-ray spectroscopy (EDX)
   2.2. Grazing-incidence wide-angle X-ray scattering (GIWAXS)
   2.3. Scanning electron diffraction (SED)
   2.4. X-ray Photoelectron Spectroscopy (XPS) and ultraviolet photoelectron spectroscopy (UPS)
3. Temperature-dependent Raman spectroscopy and GIWAXS
   3.1. Temperature-dependent GIWAXS
   3.2. Temperature-dependent Raman spectroscopy
4. Computation
   4.1. Lattice contribution to thermal conductivity
5. Thermoelectric characterizations and analysis
   5.1. Thermoelectric analysis by regular and molecular Wiedemann-Franz laws
   5.2. Summary of electrical conductivity, lattice thermal conductivity, and thermoelectric performance
   5.3. Carrier transport analysis with mobility edge, variable-range hopping, heterogeneous transport, and semi-localized transport models
   5.4. Carrier transport analysis with Boltzmann transport equation
   5.5. Influences of impurities in BHT on thermoelectric properties
6. Magnetotransport characterizations and analysis

III. Supplementary References

Supplementary Methods

1. Materials and device methods

1.1. Materials

Several batches of benzenehexathiol (BHT) have been used in this work and they were synthesized as reported [1]. Chloroform (CF), containing ethanol as stabilizer, ACS reagent, ≥ 99.8%, and Copper(II) acetate, 99.99% trace metals basis, were purchased from Sigma-Aldrich. Chlorobenzene (CB), > 99.9%, was purchased from ROMIL Ltd. Substrates of Corning EAGLE XG glass and front side-polished bare Si wafers (flat: SEMI Std.) were purchased from Corning Inc. and Active Business Company GmbH respectively. Specially designed, commercially available silicon-based Linseis chips for thermal conductivity measurement were ordered from Linseis Messgeräte GmbH. SiN_x grids with a 30 nm-thick, low-stress amorphous Si_3N_4 membrane window (NT025X) for electron diffraction measurement were ordered from Norcada Inc. The sample, from which the non-van-der-Waals layered structure of Cu_3BHT was found [2], was provided by Jinhu Dou and Chichao Pan (Peking University).

1.2. Device methods

For initial electrical conductivity and Seebeck coefficient measurements, a four-parallel-electrode device architecture was used (**Figure S1**). For all temperature-dependent measurements – electrical conductivity, Seebeck coefficient, DC and AC Hall effects, and magnetotransport – a multifunctional, integrated device architecture [8,9] was employed (**Figure S2**). For thermal conductivity measurements, commercially available silicon-based chips (referred to as Linseis chips) which can be ordered from Linseis Messgeraete GmbH Vielitzerstr (Distributor: Gammadata UK Limited) were employed (see (**Figure S3** for device architecture).

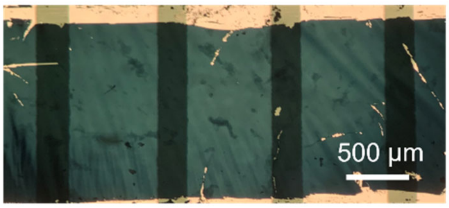

Figure S1. Optical image for the real devices with four-parallel-electrode architecture for initial sample optimalization.

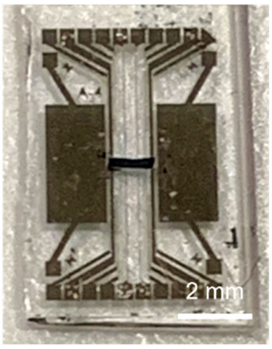

Figure S2. Optical image for the real devices with multifunctional Hall bar architecture for temperature-dependent measurements of transport coefficients.

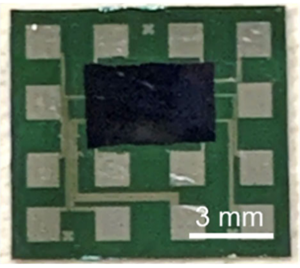

Figure S3. Optical image for the real devices based-on Linseis chips for thermal conductivity determination.

Supplementary Discussion

2. Chemical and structural characterizations

2.1. Scanning electron microscopy (SEM) and energy dispersive X-ray spectroscopy (EDS)

Through probing the cross section of the films, elements Cu, S, C, and unexpectedly O were detected, as shown in **Figure S4** (C is not shown due to the strong background signal). Ideally, Cu/S atomic ratio should be 0.50. However, the atomic ratios of Cu/S were found to be 0.54 ± 0.007, 0.57 ± 0.01, 0.58 ± 0.01, and 0.61 ± 0.02 for the samples synthesized with Cu/S loading atomic ratios of 0.33, 0.58, 0.83, and 1.08, respectively (corresponding to Cu/BHT mole ratios of 2, 3.5, 5, and 6.5). These results indicate under all growth conditions used the composition of the films remains Cu-rich and suggest the majority of chemical defects are BHT vacancies, which may be filled with the acetate anions of the metal precursor ($CH3COO^-$). The error bars for Cu/S atomic ratio represent the standard deviation of the experimental results taken at different local positions in the same sample.

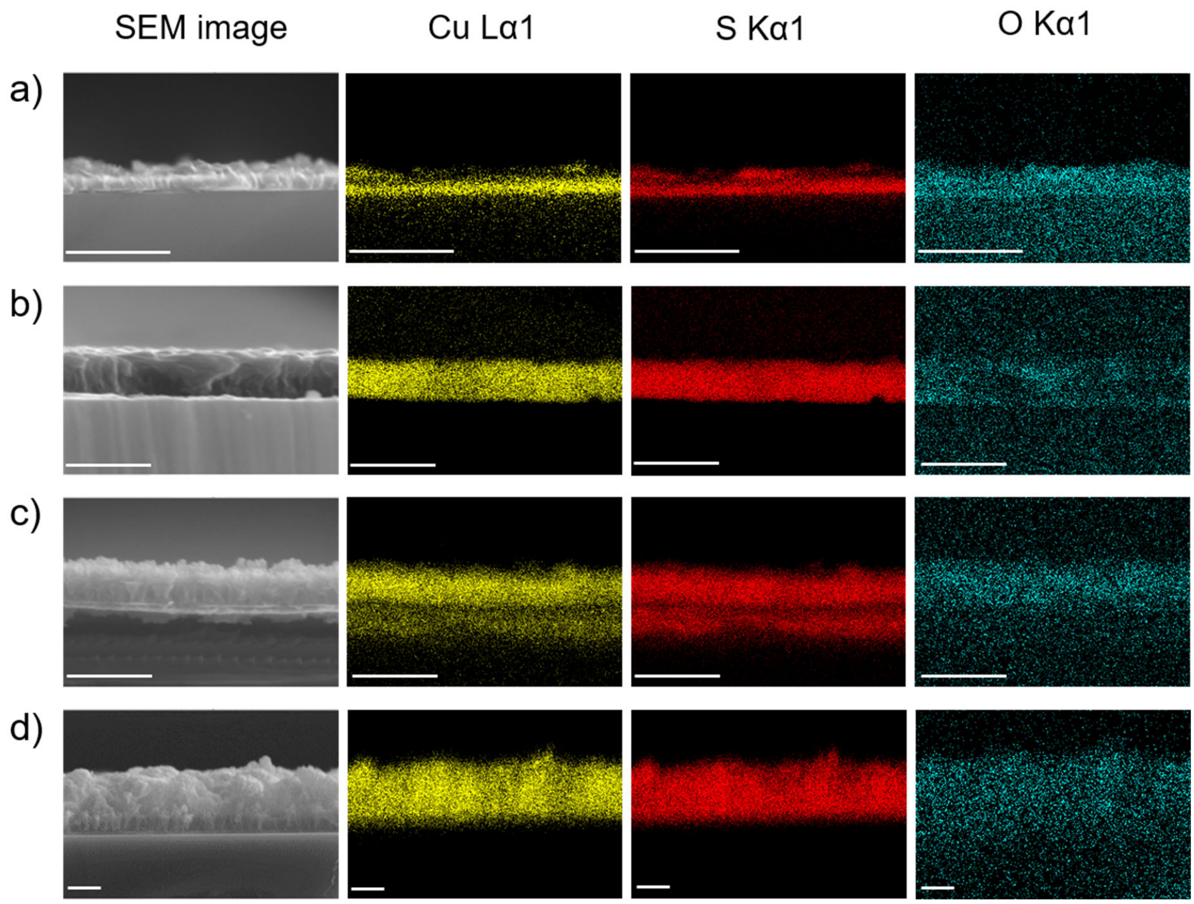

Figure S4. SEM-EDS analysis of Cu-BHT films with different compositions (different degree of chemical defects). **a – d**, Cu/BHT mole ratio (loading) of 2, 3.5, 5, and 6.5 respectively. All scale bars here are 1 um.

2.2. Grazing-incidence wide-angle X-ray scattering (GIWAXS)

To determine the structure of the Cu-BHT films prepared in this work, we performed first-principles simulations on the old AA stack [1,3,11] and the newly reported non-van der Waals layered structure [2] of Cu_3BHT. **Figure S5c,d** shows the simulated result with face-on orientation and **Figure S5e,f** shows the simulated result with all orientations averaging. Detailed assignment for the diffraction peaks can be found in **Figure S6a,b**, where the distinct peaks between the two structures are highlighted by the orange boxes. The experimental GIWAXS images of our films are much more similar to the newly reported non-van der Waals layered structure than the old AA stack, since the distinct diffraction peaks that can be assigned as (102) and (21-3) and those locate at q_z = 0.6 – 1.2 Å^{-1} and q_r = 0.6 – 2.8 Å^{-1} are seen (**Figure S6c**). Notably, in our crystalline Cu-BHT films, there is ~20% probability to exhibit triple peaks and ~80% probability to exhibit double peaks in q_z direction (**Figure S7a**), which is reproducible in two batches of films. These peaks well match with the (002), (102) and (10-2) diffraction peaks of the newly reported non-van der Waals layered structure, and in contrast the old AA stack only shows a single peak in q_z direction in simulation (**Figure S7c**).

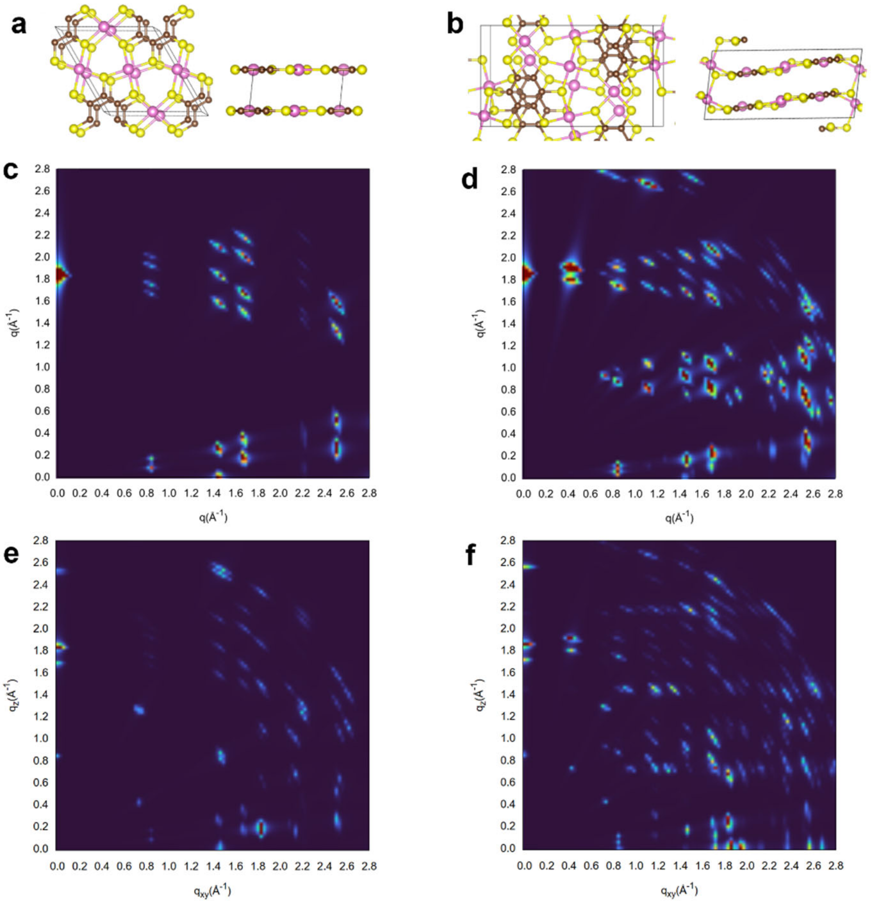

Figure S5. GIWAXS simulation of the old AA stack and the newly reported non-van der Waals layered structure of Cu_3BHT. **a,b**, Unit cells of the two structures. **c,d**, Simulated GIWAXS images with face-on orientation, i.e. with layers in parallel to the substrate. **e,f**, Simulated GIWAXS images with all orientations averaging without preference. The left and the right panels are for the old AA stack and the newly reported non-van der Waals layered structure, respectively.

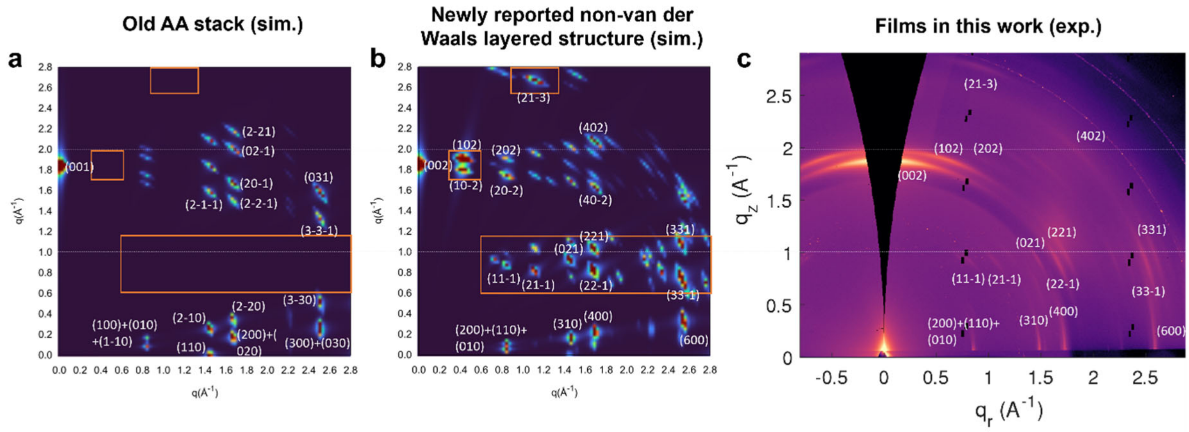

Figure S6. Comparison between the simulated GIWAXS images for the old AA stack and the newly reported non-van der Waals layered structure of Cu_3BHT and the experimental GIWAXS image of our crystalline Cu-BHT films. **a**, Simulation of the old AA stack. **b**, Simulation of the newly reported non-van der Waals layered structure. **c**, Experimental GIWAXS image (Cu-BHT ratio of 2) in this work. The distinct diffraction peaks used for structural determination are highlighted by the organic boxes.

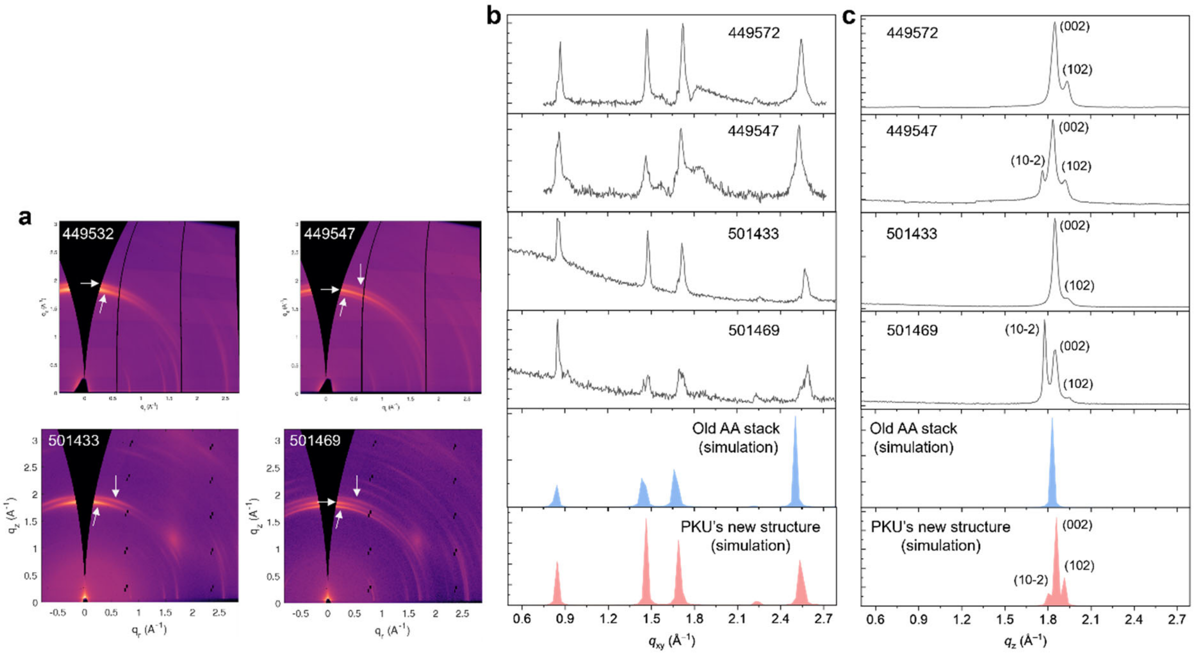

Figure S7. Comparison of the diffraction line profiles on q_{xy} and q_z directions between simulation of the old AA stack and the newly reported non-van der Waals layered structure and experiment. **a**, Selected representative experimental GIWAXS images that show double or triple peaks in q_z direction. **b**, Line profiles on q_{xy} direction. **c**, Line profiles on q_z direction.

We find that lattice strain is present in our Cu-BHT films, and the level can have region-to-region and sample-to-sample variations. There is also a probability of 20% for the measurement to yield a larger spacing of $d_{(200)}$ = 7.94 ± 0.02 Å for crystalline samples of Cu/BHT ratio 2 (**Figure S8**), with respect to the reported value of 7.35 – 7.38 Å in the literature [1,3,11,25]. Such a lattice parameter variation is believed to be generated by the presence of defects (vacancies). No direct evidence can be found for supporting the presence of another phase since both X-ray and electron diffractions as well as Raman spectra have never revealed a different pattern and, only small changes in spacing (~8%) or frequency (< 2 cm^{-1}) at

a given Cu/BHT ratio can be observed. Corresponding discussion on electron diffraction can be found in **Section 2.3**.

In order to gain insight into the ordering of materials, X-ray diffraction has been widely used with Scherrer **Equation (S1)**, and **Equation (S2)**, for estimating the coherence length L_c and the paracrystalline disorder g respectively.

$$L_c = \frac{2\pi K}{\Delta_q} \tag{S1}$$

$$g = \frac{\sqrt{\Delta_q d_{hkl}}}{2\pi} \tag{S2}$$

K is a shape factor (typically 0.8 – 1), Δ_q is the full width at half maximum (FWHM) of a diffraction peak, and d_{hkl} is the lattice plane separation. However, these two equations are in two extremes: the Scherrer equation assumes crystallite size is the dominating effect to peak broadening (with lattice disorder being ignored) while the latter takes just cumulative lattice disorder into account to determine the paracrystallinity [26,27]. Consequently, these two methods analyzing an individual diffraction peak provide inaccurate estimates when both lattice disorder and finite size effects are present, which both lead to peak broadening.

Crystallite size effects are not dependent on diffraction order whereas lattice distortion is. Lattice distortion can manifest itself in non-cumulative disorder, for example, due to thermal fluctuations that are usually characterized by lowering peak intensity, and in cumulative disorder i.e. paracrystalline disorder g) [26–28] in which variation in local atomic displacements cause the crystallite to lose coherence with the ideal lattice over long length scales. In addition, inhomogeneous broadening of diffraction peaks resulted from such as lattice parameter variation e_{rms} due to nonuniform strain, can also lead to order-dependent broadening. These different mechanisms of peak broadening can be deconvoluted through line shape and line width analysis as a function of diffraction order. Therefore, diffraction order-dependent line shape and line width analysis is performed according to methods developed in refs [26–28].

To obtain an estimation of the relative contributions from different types of disorder, a pseudo-Voigt function, which is a linear combination of a Gaussian function (G) and a Lorentzian function (L) in a form of ηL + (1 − η)G [26], is first employed to model a series of in-plane diffraction peaks (h00). The results are summarized in **Figure S10g, h**. The pseudo-Voigt mixing parameter η contains more subtle information about peak shape than peak width alone, with values approaching to one (Lorentzian peak shape) point to a paracrystallinity-dominated regime and values approaching to zero (Gaussian peak shape) suggest that strain-induced lattice parameter fluctuations dominate with little effect from paracrystallinity [26], although may also appear more complex if the distribution of strain within crystallites is not Gaussian-distributed. For instance, in some samples we observe lattice strain fluctuations resulted from positional defects (vacancies and dislocations), which manifest as asymmetric and split diffraction peaks, leading to a non-gaussian distribution of crystallite strain within the sample, as illustrated in **Figures S8, S9** and discussed in the first two paragraphs of this section. Nonetheless, in all samples the observed Lorentzian lineshapes and order-dependent broadening indicate ordering in Cu-BHT is dominated by paracrystalline displacement (positional disorder) with a smaller contribution from strain-induced lattice spacing fluctuations.

To gain meaningful insight and to quantify the different types of disorders, a more accurate fast Fourier transform (FFT) algorithm first developed by B. E. Warren and B. L. Averbach [28] is performed to the films of Cu/BHT ratio of 2. This can be done in two ways: WA full-fit[26] and WA graphical analyses [27]. To start with, the first step is to correct the X-ray profile for the effects not reflecting the internal structure of the material. After correction and background subtraction, the nth-order Fourier cosine transform coefficient of a specific h-order diffraction peak has a form of

$$A_h(n) = A^S(n) A_h^e(n) A_h^D(n) \tag{S3}$$

$$L(n) = n/N\Delta q \tag{S4}$$

where $A^S(n)$, $A_h^e(n)$, and $A_h^D(n)$ are the normalized Fourier cosine coefficients for crystallite size, lattice parameter fluctuation, and displacement disorder, respectively. The Fourier coefficients with equivalent unit cells apart $n = -N/_2, -N/_2 + 1, \dots, N/_2$ contain information on a length scale $L(n)$ dependent on angular distance between scattering points Δq and number of scattering data points N. For small disorder fluctuations the three normalized Fourier cosine coefficients can be approximated and further written as [27]:

$$A^S(n) = \frac{N(n)}{N_3} \quad \text{(S5)}$$

$$A_h^e(n) = \langle \cos(2\pi hne) \rangle \approx e^{-2\pi^2 h^2 n^2 e^2} \quad \text{(S6)}$$

$$A_h^D(n) = \langle \cos(2\pi h Z_n) \rangle \approx e^{-2\pi^2 h^2 \langle Z_n^2 \rangle} \quad \text{(S7)}$$

$$\langle Z_n^2 \rangle = ng^2 \quad \text{(S8)}$$

where $N(n)$ is the average number of crystal cells $L(n)$ apart per column of cells. $\langle e^2 \rangle$ is the lattice parameter fluctuation and $\langle Z_n^2 \rangle$ describes the displacement between two unit cells within a column that is n unit cells apart. By combining above equations, finally, one can separate the size and the distortion effects by taking a form of natural logarithm [27]:

$$\ln A_h(n) = \ln \frac{N(n)}{N_3} - 2\pi^2 h^2 n f(n) \quad \text{(S9)}$$

$$f(n) = g^2 + \langle ne^2 \rangle \quad \text{(S10)}$$

By plotting $\ln A_h(n)$ against h^2 with holding n as constant, one can determine the terms of $\ln \frac{N(n)}{N_3}$ and $-2\pi^2 n f(n)$ from the intercept at y axis and the slope respectively. Thereafter the effects of paracrystalline disorder, g, and lattice parameter fluctuation, $\langle e^2 \rangle$ (also termed as e_{rms}), can be further separated by plotting $f(n)$ against n. This is WA graphical analysis methodology [27].

Similarly, WA full-fit analysis[26] can be performed within the same theoretical model. It can be directly performed on the normalized Fourier cosine coefficients by using **Equations (S9), (S10)**. Both methods are applied to our material system with Cu/BHT ratio of 2. Corresponding plots are shown in **Figure S10i, j** for the full-fit method and **Figure S10k, l** for the graphical method. g, e_{rms}, and L_c are extracted to be 3 – 4%, < 1%, and 31.4 ± 1.7 nm, respectively, for Cu/BHT ratio 2, indicating paracrystalline disorder dominates but strain-induced lattice parameter variation still provides a non-negligible contribution to the observed diffraction data.

Due to the fact that the peak broadening of other compositions is sufficiently large, falling within the range of strongly disordered materials, **Equation (S2)** is instead reasonably used to simplify the analysis process [26], and g values are found to gradually increase from 10 to 13% as Cu/BHT ratio increases. For strongly disordered materials, the X-ray coherence is sufficiently hindered by disorder, not finite size, so the determination of crystallite size L_c is difficult. Instead, a disorder-associated coherence length ξ[26] (in the absence of lattice spacing fluctuations) expressed as

$$\xi = \frac{d_{hkl}}{2\pi g^2} \quad \text{(S11)}$$

is used and found to be no larger than 10 nm for all the rest samples.

All error bars in structural analysis from diffraction data (e.g. **Figures 2e,f, S10**) reflect the uncertainties of the values due to the fit of the diffraction peaks.

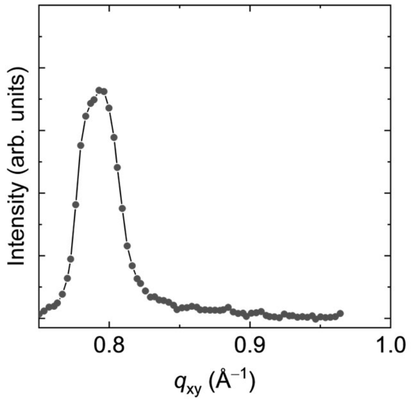

Figure S8. GIWAXS data of a film showing smaller reciprocal spacing (larger lattice spacing) than usual in the in-plane direction. Corresponding GIWAXS image is **Figure S10a**. This in-plane profile showing (200) at q_{xy} = 0.79 Å^{-1} ($d_{(200)}$ = 7.94 Å) means that the lattice scales up by 1.08 in real space with respect to the values of $d_{(100)}$ = 7.35 – 7.38 Å extracted from the literature [1,3,11,25]. The spacing of this profile is also different from that of **Figure S9** even though both were synthesized with identical method at same Cu/BHT ratio of 2, indicating a presence of lattice parameter variation in Cu-BHT films.

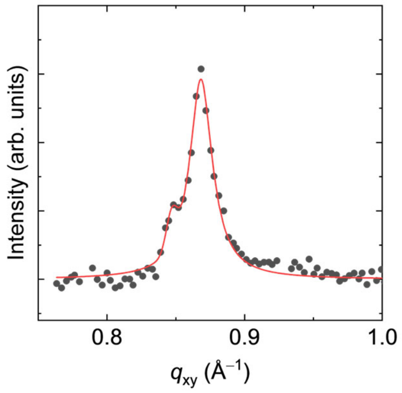

Figure S9. A diffraction peak (200) at q_{xy} = 0.86 Å^{-1} ($d_{(200)}$ = 7.31 Å) extracted from another GIWAXS image of Cu/BHT ratio of 2.

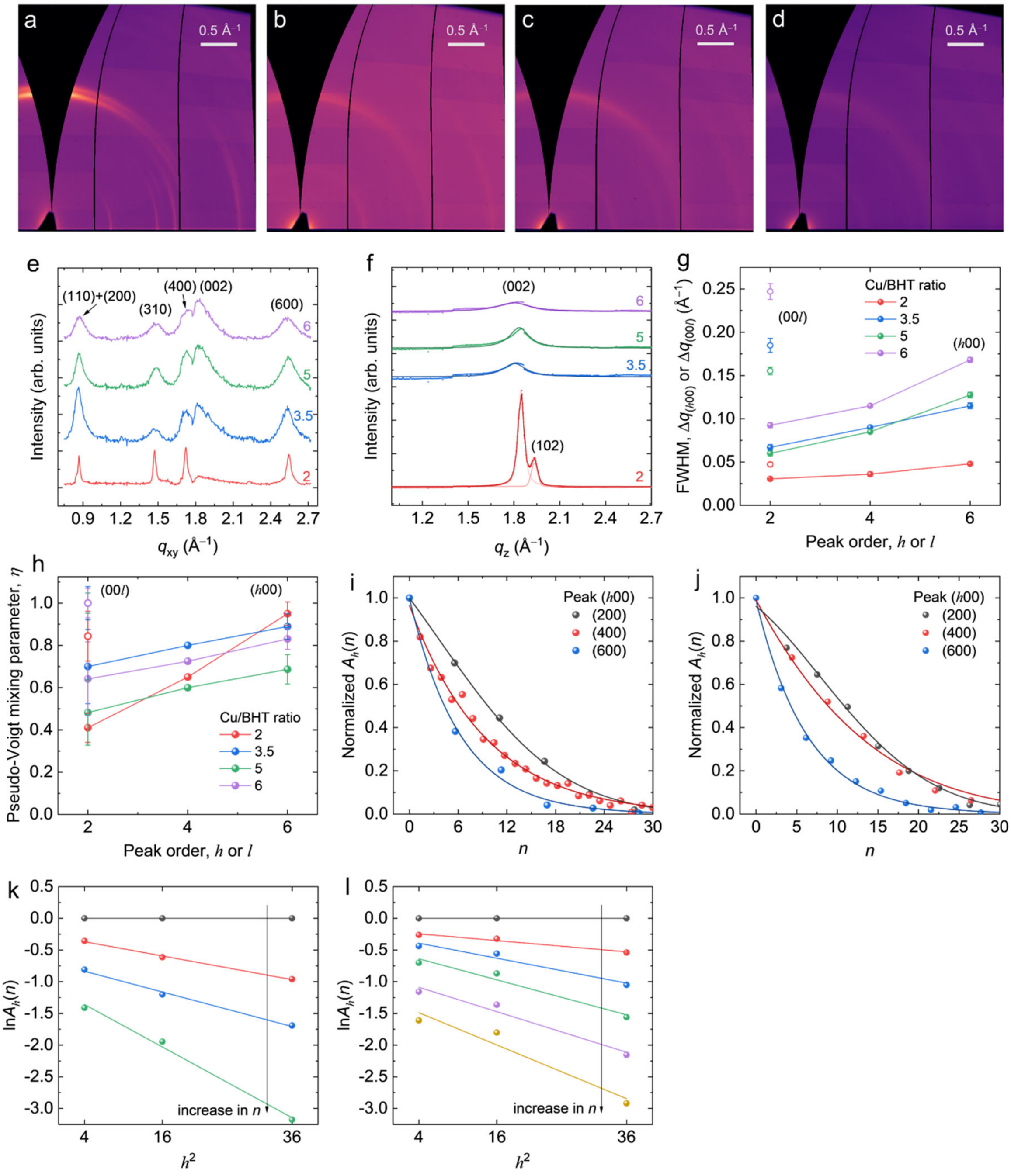

Figure S10. Structural (dis)order analysis of Cu-BHT films by GIWAXS. **a – d**, GIWAXS images for Cu/BHT mole ratio of 2, 3.5, 5, and 6, respectively. **e** and **f**, In-plane and out-of-plane profiles extracted from images a – d, clearly showing that diffraction peak (002) is along the q_z direction while peaks (200), (400), and (600) are along the q_{xy} direction, so that the layered structure of the Cu-BHT films is identified to be parallel to the substrate, i.e. face-on orientation. **g** and **h**, FWHM Δ_q and the Pseudo-Voigt mixing parameter η, in both (h00) and (00l) obtained by fitting the extracted diffraction peaks. The solid and the open circles represent (h00) and (00l). **i** and **j**, Fourier transform coefficients A_n of isolated peaks (h00) of Cu/BHT ratio 2 processed by WA full-fit. **k** and **l**, Fourier transform coefficients A_n of isolated peaks (h00) of Cu/BHT ratio 2 processed by WA graphical method. The plots in **i** and **k**, and the plots in **k** and **l** are for two individual samples and they both exhibit same results.

2.3. Scanning electron diffraction (SED)

To ensure the films are thin enough and thus electron-transparent, a thickness of about 50 – 70 nm for Cu/BHT ratio 2 was used in this experiment. The SEM image of **Figure S11a** shows the surface morphology of the sample where flakes sitting flat (in green) and flakes with their edges facing up (in red) can be found. These two kinds of features correspond to the red and the green boxes labelled in the right panel of **Figure S11b**. Since virtual dark-field (vDF) mode only allows scattered electrons to transmit whereas unscattered electrons is excluded from the aperture, the dark areas mean the areas where not much electron scattering happening while the bright areas (such as the regions of the red and the green boxes) are indicative of the areas with ordered and crystalline features at which electrons get scattered strongly. The diffraction pattern in the red box shows diffraction peak (002) found at 0.275 ± 0.015 $Å^{-1}$ while that the green box exhibits diffraction peak (200) found at 0.126 ± 0.015 $Å^{-1}$. Similarly, same results were found for Cu/BHT ratio 3.5 (**Figure S12**). By acquiring and indexing each diffraction pattern in the scanned area an orientation map (i.e. **Figure 1h,i**) can be computed. Some representative diffraction images showing the in-plane lattice are further added as **Figure S13** as examples. These lattice spacings match with the in-plane spacing of the non-van der Waals layered Cu_3BHT[2].

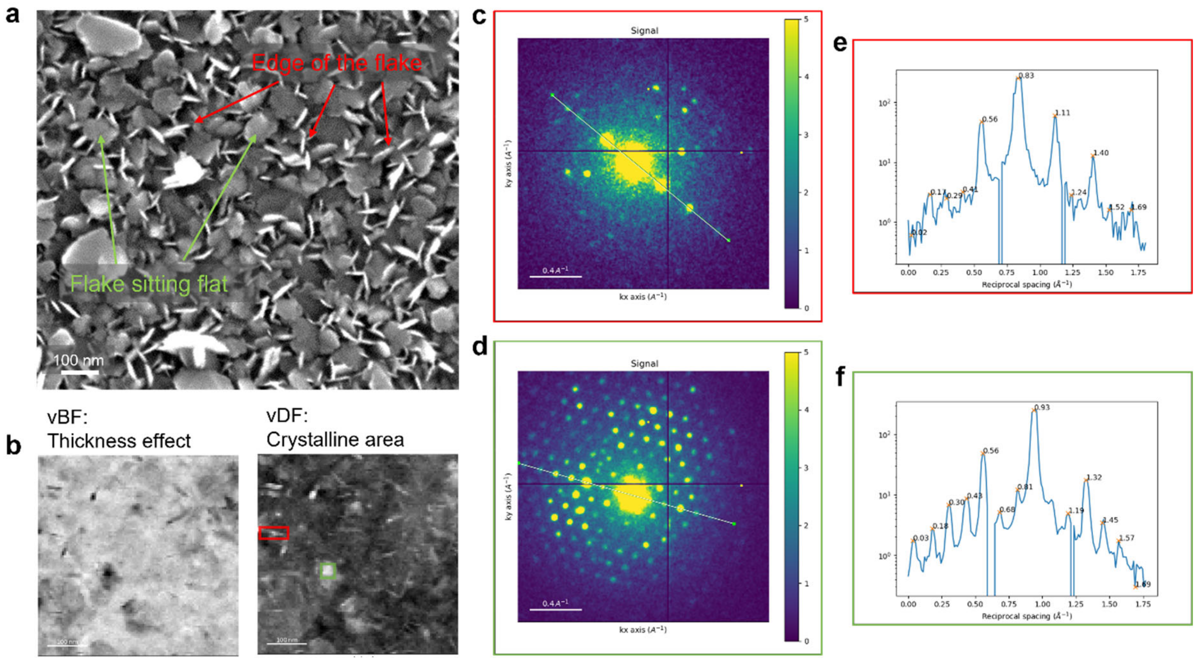

Figure S11. SED experiment on Cu/BHT ratio 2 showing spatial crystallinity distribution and representative electron diffraction patterns for edge-on and face-on grain orientations. **a**, Surface morphology of the sample taken by SEM. **b**, Virtual bright-field (vBF) and virtual dark-field (vDF) images taken by SED. **a**, **b**, and **Figure 2h,i** in the main text are not showing an identical region, but the sample is a same one. **c** and **d**, edge-on and face-on electron diffraction patterns taken at the red and the green boxes labelled in **b**. Edge-on orientation means zone axes [200] and [020] normal to the substrate while face-on means zone axis [002] normal to the substrate. **e** and **f**, Diffraction profiles in reciprocal spacing correspond to the lines in **c** and **d** respectively.

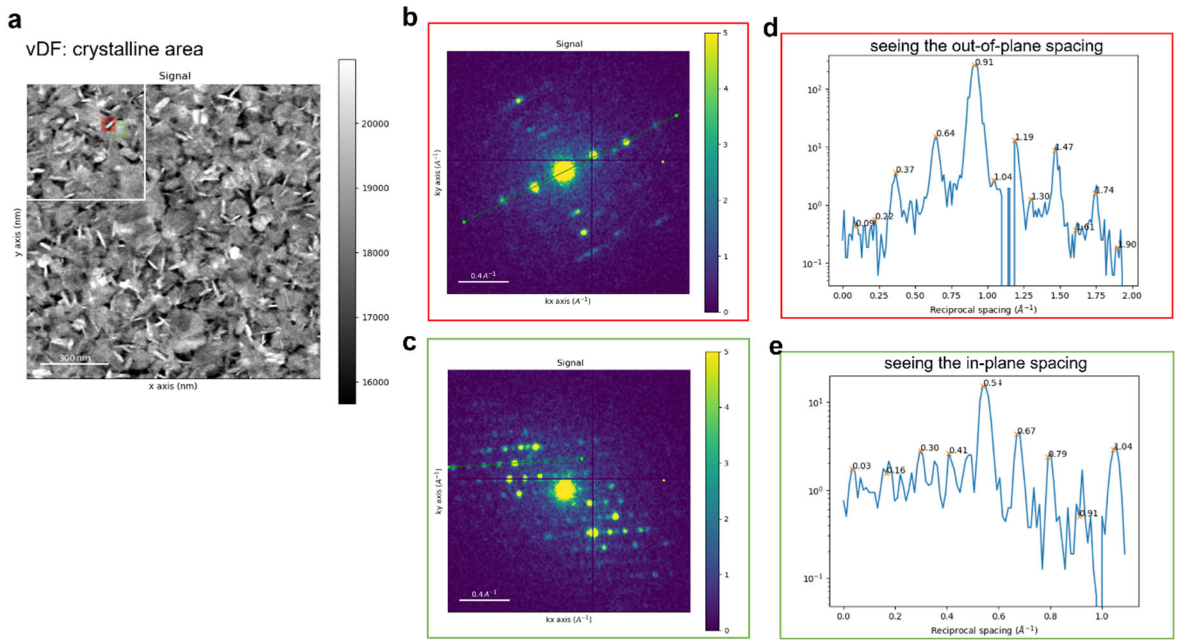

Figure S12. SED experiment on films of Cu/BHT ratio 3.5. **a**, vDF images taken by SED. **b**,**c**, Representative edge-on and face-on electron diffraction patterns taken at the red and the green boxes labelled in **a**. **d**,**e**, Diffraction profiles in reciprocal space correspond to the lines in **b** and **c** respectively.

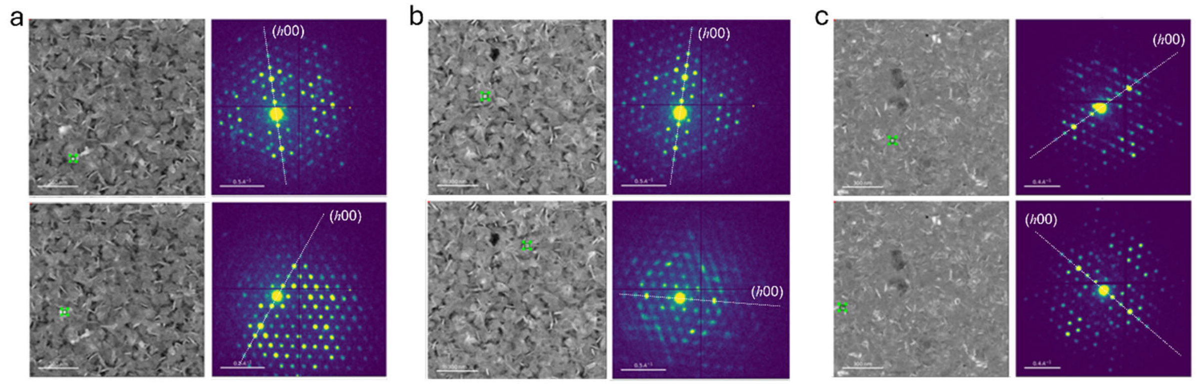

Figure S13. Electron diffraction images from SED measurements of films of Cu/BHT ratio 3.5. **a**, **b**, **c** are different regions. They all show a lattice matched with the in-plane lattice of the non-van der Waals layered Cu_3BHT. The orientations of the grains is predominantly face-on with minor tilts of the zone axis with respect to the substrate normal.

2.4. X-ray Photoelectron Spectroscopy (XPS) and ultraviolet photoelectron spectroscopy (UPS)

XPS experiment was performed on Cu-BHT films prepared by different Cu/BHT ratios and they all show spectra essentially the same as what the literature reported for Cu_3BHT[16,18] (**Figures S14,S15**).

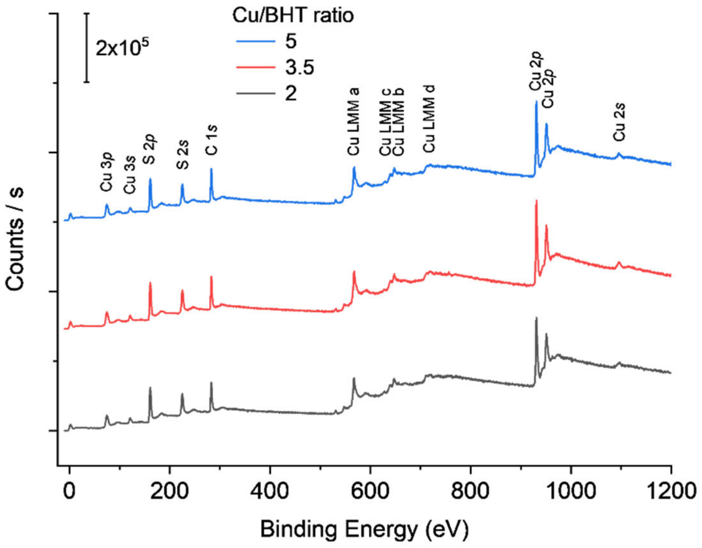

Figure S14. XPS spectra of Cu-BHT films prepared by different Cu/BHT ratios. **a**, Cu/BHT ratio 2. **b**, Cu/BHT ratio 3.5. **c**, Cu/BHT ratio 5.

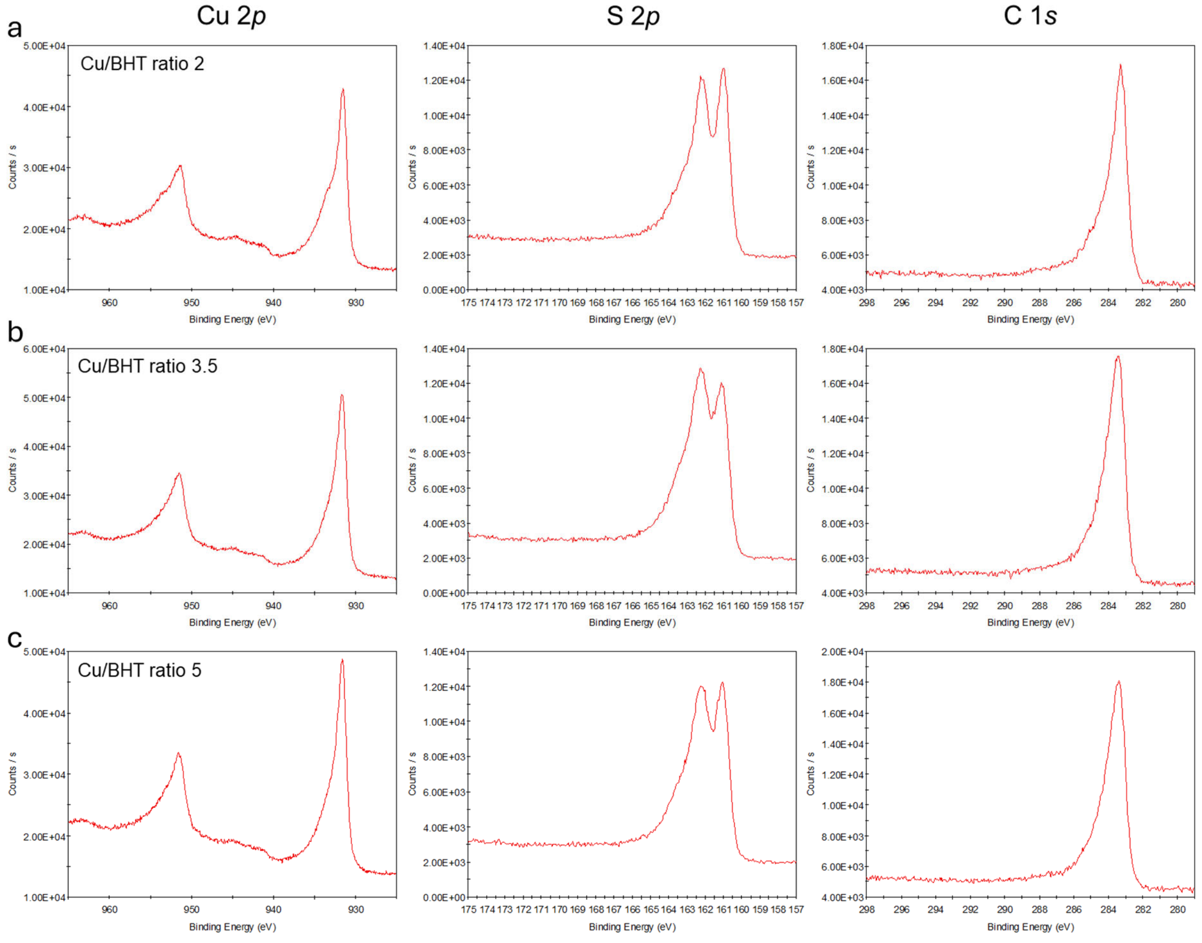

Figure S15. High-resolution XPS Cu 2*p*, S 2*p*, and C 1*s* spectra of Cu-BHT films prepared by different Cu/BHT ratios. **a**, Cu/BHT ratio 2. **b**, Cu/BHT ratio 3.5. **c**, Cu/BHT ratio 5.

During UPS measurement the samples were biased by -5 eV to shift the spectra away from the influences of the spectrometer's own work function as well as increases count rates particularly for secondary electrons. The spectra in **Figure S16** have been energy corrected.

Strong secondary tails and high-resolution expansions of the Fermi edge are shown below. Measurements of the positions of the Fermi edge E_{onset} and the secondary tail E_{cutoff} allow the calculation of the work function Φ of the Cu-BHT films by using **Equation (S12)**:

$$\Phi = hv - (E_{cutoff} - E_{onset}) \tag{S12}$$

where hv is the source energy of the photons which is 21.22 eV for HeI source. The work functions for the crystalline Cu/BHT ratio 2 and the amorphous ratio 5 are extracted to be 4.91 and 4.99 eV respectively.

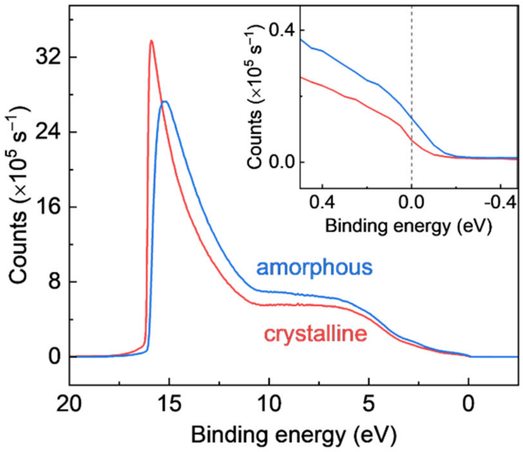

Figure S16. UPS spectra of a crystalline film with Cu/BHT ratio of 2 and an amorphous film with Cu/BHT ratio of 5. Work functions are found to be 4.91 and 4.99 eV for ratio 2 and ratio 5 respectively.

3. Temperature-dependent Raman spectroscopy and GIWAXS

3.1. Temperature-dependent GIWAXS

Temperature-dependent GIWAXS measurement can provide insights into lattice behavior upon thermal excitation. Uniaxial thermal expansion coefficient $\alpha_{a,b,c}$ can be evaluated by $\alpha_i \Delta T = \Delta L_i / L_{i0}$ where ΔL_i is the change in length of sample due to heating or cooling and L_{i0} is the original length of the sample at room temperature. For structural ordering as a function of temperature, the theory, analysis method, and data processing can be found in **Section 2.2** for details.

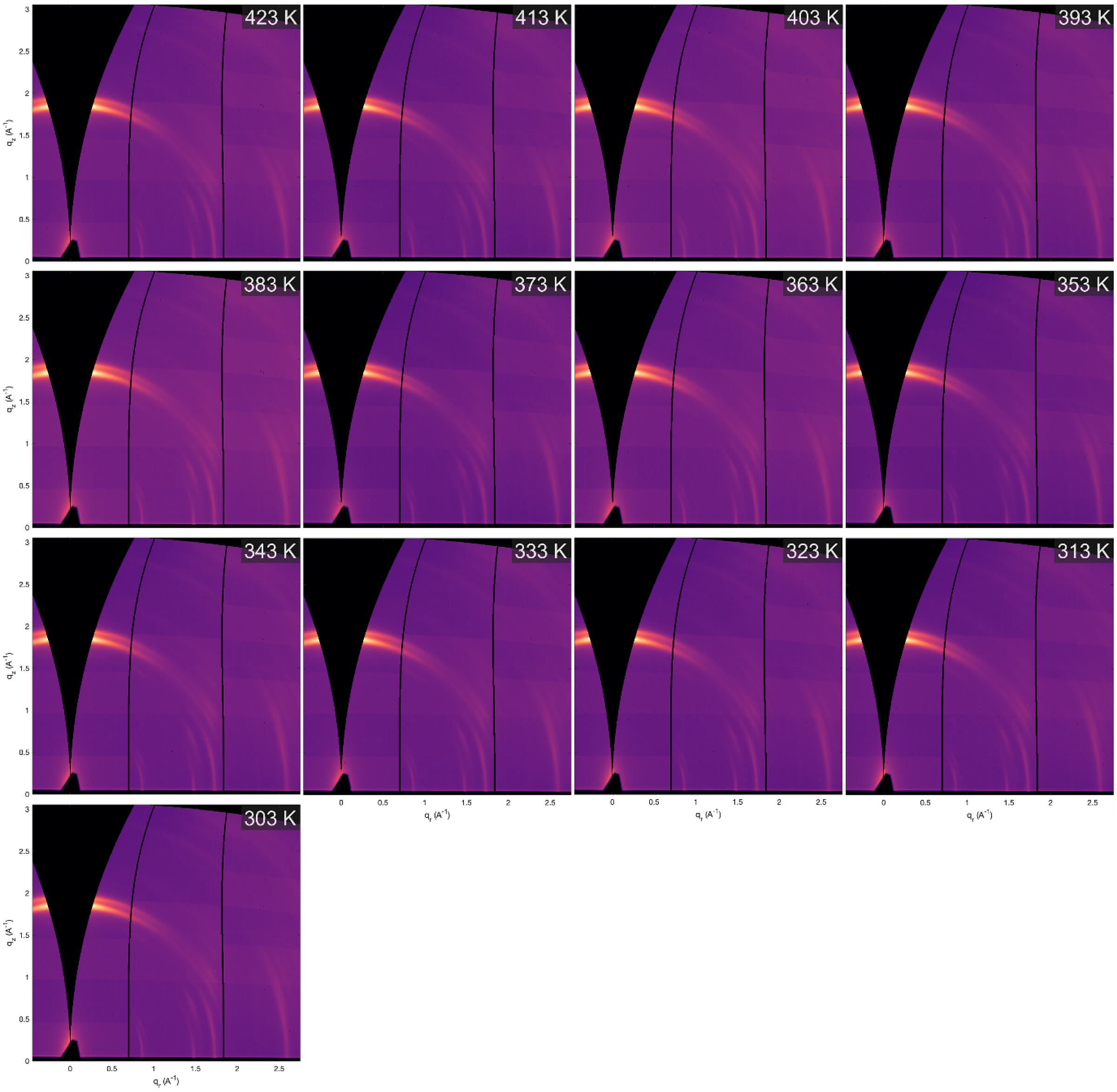

Figure S17. GIWAXS images for a Cu-BHT film synthesized with Cu/BHT ratio 2. Images were collected from 423 to 303 K in steps of 10 K.

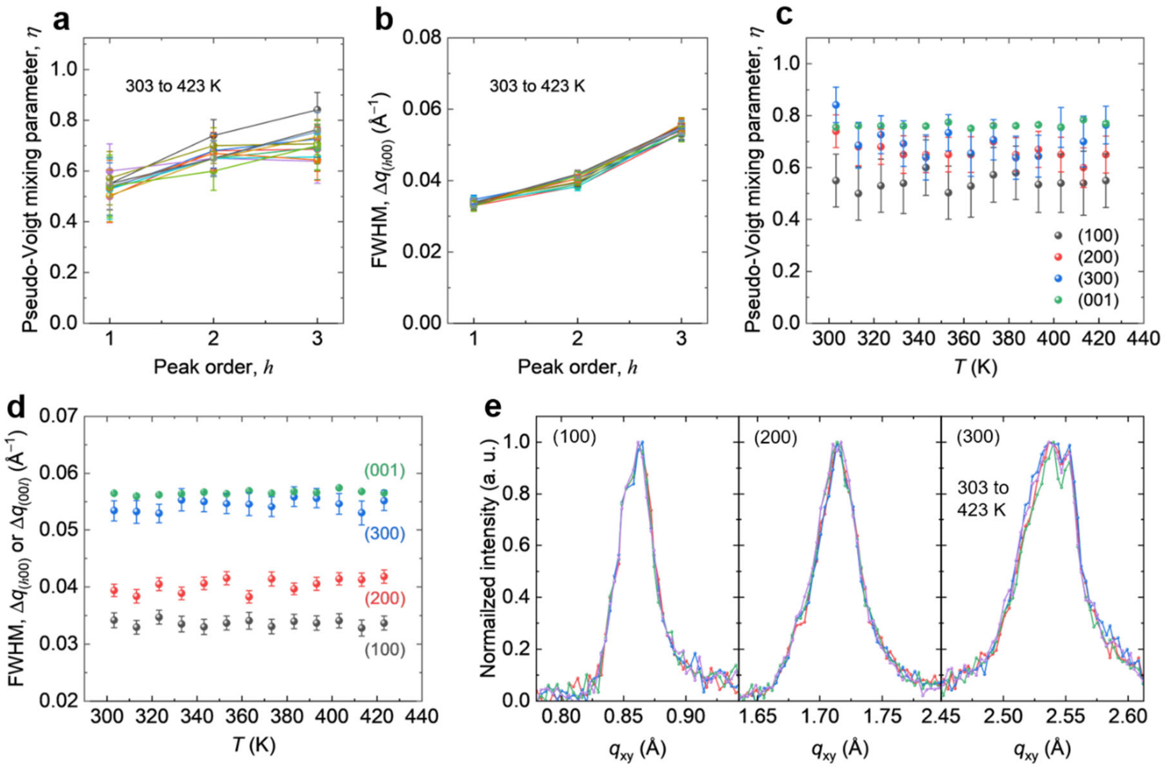

Figure S18. Temperature-dependent structural and ordering characteristics extracted from **Figure S17**. **a** and **b**, Pseudo-Voigt mixing parameter η and FWHM Δ_q against peak order h respectively. **c** and **d**, Plots of η and Δ_q against temperature. **e**, Peaks (h00) as a function of temperature presenting a temperature-insensitive nature. The theory, analysis method, and data processing can be found in **Section 2.2** for details. All error bars here reflect the uncertainties originating from the fitting of the diffraction peaks when extracting the peak positions and full widths of half maximum.

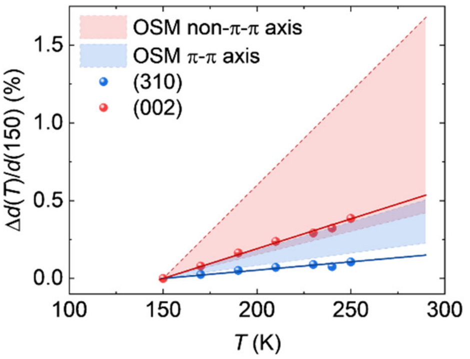

Figure S19. Changes of lattice parameters as a function of temperature from 150 to 290 K. The data are extracted from **Figure 4c,d**. Diffraction peaks (002) and (310) are used to represent the changes in the in-plane and the out-of-plane directions. From the slope of the data thermal expansion can be extracted. The light orange and the light blue areas are the usual ranges for the non-π-π axis and π-π axis of organic semiconductors. These results show that in all lattice directions our Cu-BHT films have much weaker thermal expansion behaviors than the soft organic semiconductors.

3.2. Temperature-dependent Raman spectroscopy

In addition to GIWAXS, Raman spectroscopy was used to justify that the use of the newly reported Cu_3BHT structure in this work is the most suitable. We measured the Raman spectra of our films and the sample from which the new Cu_3BHT structure was found. The comparison in **Figure S20a** shows that they have identical Raman pattern, and the difference is the intensities of the peaks at 470.3 and 495.6 cm^{-1}. Such a difference in the intensity of the two peaks was also found in our films of Cu/BHT ratio of 2 (**Figure S20c**), depending on the regions where the measurements took place. These results reasonably reflect that our crystalline samples have the same structure as the newly reported Cu_3BHT, and the difference in the intensity of the two peaks is likely due to different gain orientations.

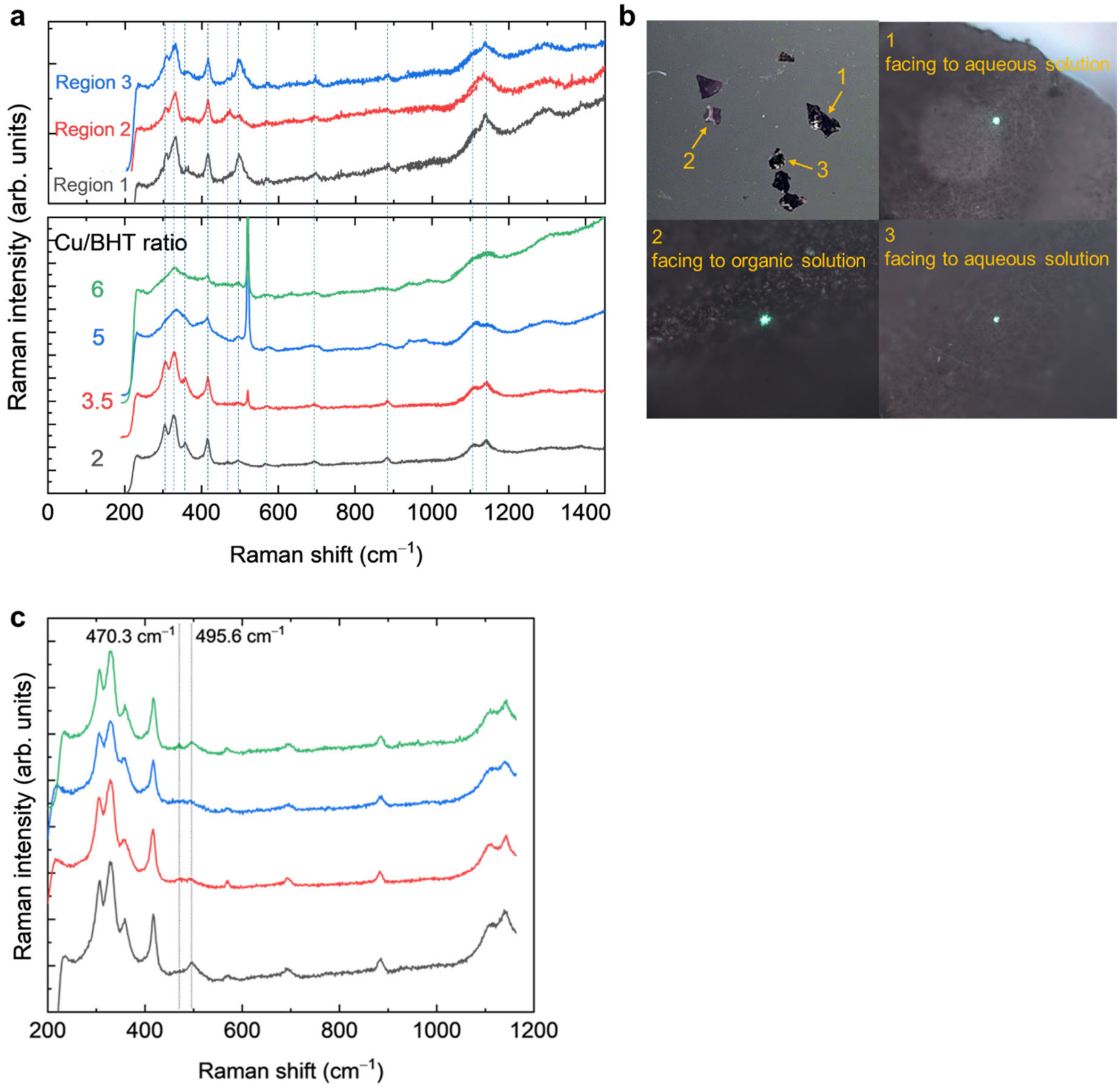

Figure S20. Comparison of the Raman spectra of our films with the sample from which the new Cu_3BHT structure was determined. **a**, Top: Raman spectra of the sample of the new Cu_3BHT structure, regions 1, 2, and 3 indicate different measurements performing on films labelled as 1, 2, and 3 in **b**; bottom: Raman spectra of our films with different Cu/BHT ratios. **c**, Raman spectra measured on different regions of our film with Cu/BHT ratio of 2, also showing a variation of the relative intensity of the peaks at 470.3 and 495.6 cm^{-1}.

The volumetric thermal expansion coefficient α can be estimated from temperature-dependent Raman spectroscopy using the relationship between vibrational frequency ω and temperature T. The vibrational frequency at a given temperature is a sum of different contributions, which is given by **Equation (S13)** [29]:

$$\omega(T) = \omega_0 + \Delta\omega_{\mathrm{TE}} + \Delta\omega_{\mathrm{A}} + \Delta\omega_{\mathrm{M}} \quad \text{(S13)}$$

where ω_0 is the Raman shift at 0 K, $\Delta\omega_{\mathrm{TE}}$ and $\Delta\omega_{\mathrm{A}}$ are the changes in the Raman shift caused by thermal expansion and anharmonic effects respectively. $\Delta\omega_{\mathrm{M}}$ represents frequency change due to thermal expansion mismatch between sample and substrate. For bulk samples measured in this work, $\Delta\omega_{\mathrm{M}}$ is negligible in comparison to other terms. It is also reasonable to neglect $\Delta\omega_{\mathrm{A}}$ to simplify **Equation (S13)** to **Equation (S14)** [29],

$$\omega(T) = \omega_0 + \Delta\omega_{\mathrm{TE}} \quad \text{(S14)}$$

since the temperature dependences of the line shape and the line width of vibrational peaks < 500 $\mathrm{cm^{-1}}$ indicate a relatively long vibrational lifetime and harmonic nature[30]. Consequently, the most common approach to resolving anharmonic effects – quasi-harmonic approximation (QHA) in which the temperature dependence of vibrational frequencies is account for thermal expansion only[30] – is applicable here. The Grüneisen constant model describes $\Delta\omega_{\mathrm{TE}}$ as **Equation (S15)** [29],

$$\Delta\omega_{\mathrm{TE}}(T) = \omega_0 \left[\exp\left(-N\gamma \int_{T_0}^{T} \alpha \mathrm{d}T\right) - 1\right] \quad \text{(S15)}$$

where N, γ, and α are degeneracy of the Raman mode, Grüneisen parameter, and volumetric thermal expansion coefficient, respectively. The relationship between α, γ, and T is defined as **Equation (S16)** [29],

$$\alpha = (A + BT)/\gamma = c_0 + c_1 T \quad \text{(S16)}$$

By combining **Equations (S14) – (S16)**, volumetric thermal expansion coefficient at 300 K is best fitted to be 2.0×10^{-5} and 1.4×10^{-5} $\mathrm{K^{-1}}$, with Grüneisen parameter found to be 1.18 and 0.87 for $N = 1$ and $N = 2$ respectively, by using a vibrational band attributed to in-plane displacement of S atoms. These values are smaller than that of organic semiconductors [30] (~ 10^{-4} $\mathrm{K^{-1}}$) and comparable to inorganic and other 2D materials [29,31] (10^{-5} – 10^{-6} $\mathrm{K^{-1}}$, 2.5×10^{-5} $\mathrm{K^{-1}}$ for WS_2 for example), indicating Cu-BHT's less soft and more harmonic nature.

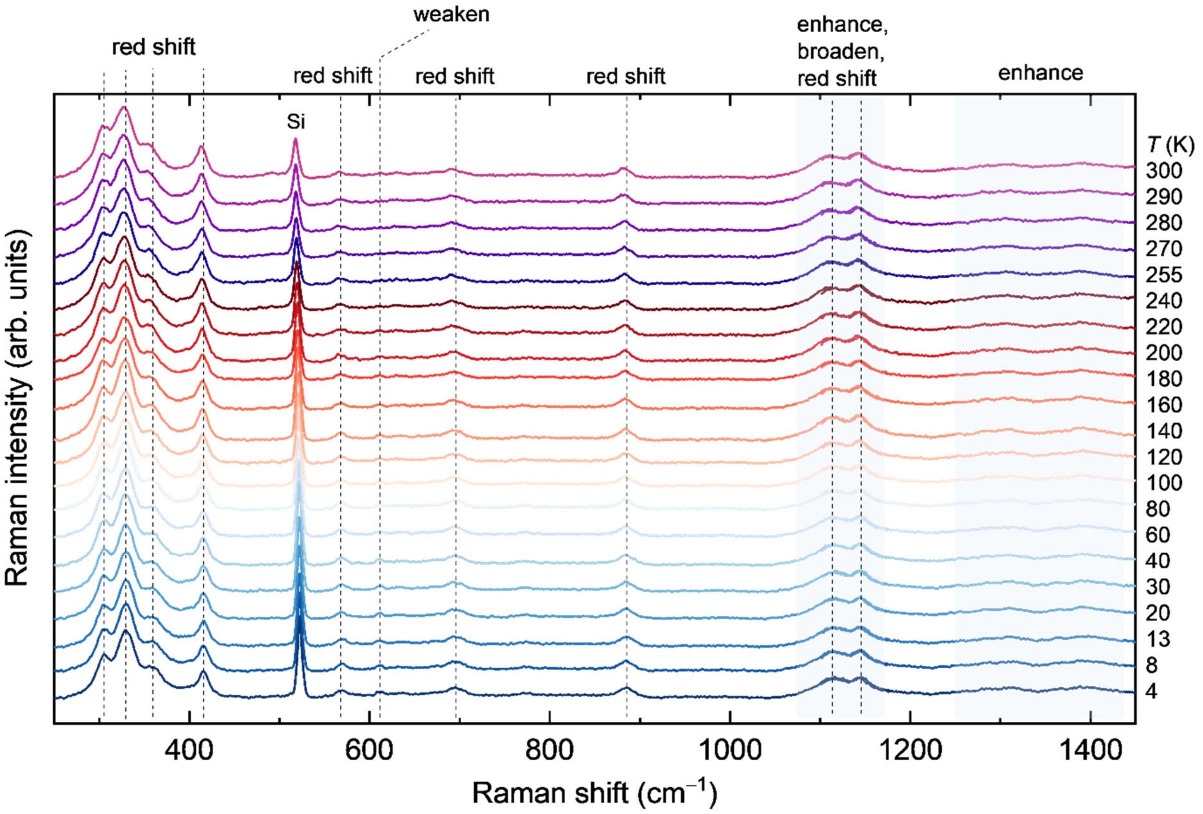

Figure S21. Temperature-dependent Raman spectra measured from 4 to 300 K with a laser excitation of 532 nm.

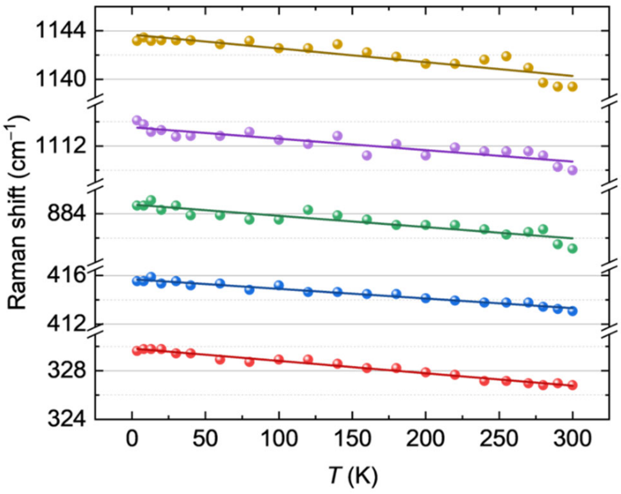

Figure S22. Linear fit of Raman shift as a function of temperature. The fitting parameters are listed in **Table S1**.

Table S1. Fitting parameters – temperature coefficient χ and vibrational frequency at 0 K ω_0 – of different vibrational modes. These values are fitted by the most common linear relationship between Raman shift and temperature: $\omega = \omega_0 + \chi T$.[29–32]

ω_{300} (cm^{-1})	Major vibration mode	ω_0 (cm^{-1})	χ (cm^{-1} K^{-1})
326.81	S atoms in-plane displacement	329.83 ± 0.06	−0.0102 ± 0.0004
413.07	S atoms in-plane displacement	415.70 ± 0.06	−0.0079 ± 0.0004
881.13	C ring in-plane deformation	884.8 ± 0.2	−0.0093 ± 0.0009
1109.99	C ring in-plane deformation	1113.5 ± 0.2	−0.009 ± 0.001
1139.39	C ring in-plane deformation	1143.7 ± 0.2	−0.011 ± 0.001

Table S2. Summary of temperature coefficients of vibrational frequency, thermal expansion, and lattice thermal conductivities of different material systems.

Material system(s)	Temperature coefficient of vibrational frequency $\lvert\chi\rvert$ [a] (10^{-2} cm^{-1} K^{-1})	Uniaxial thermal expansion coefficient $\alpha_{a,b,c}$ (10^{-6} K^{-1})	In-plane lattice thermal conductivity κ (W m^{-1} K^{-1})
Organic molecules BTBT, DNTT, and their derivatives, Rubrene	0.7 – 3.4 (diPh-BTBT and DNTT) [b]<br>0.9 – 10.1 (BTBT, diBu- and diC8-BTBT)	16 – 36 (p-p axis) [c]<br>30 – 120 (non-p-p axis) [c]	0.05 (C8DNTT)<br>0.25 (DNTT)<br>0.4 (Rubrene)
Organic polymers P3HT and PBTTT	–	–	0.35 – 0.48 (P3HT)<br>0.39 (PBTTT)
Inorganic layered materials MoS_2, WS_2	1.2	1 – 8.6	> 10
Metal-organic coordination nanosheet Cu-BHT	0.8 – 1.1 (S atoms displacement and C ring deformation, in-plane) [d]	4.0 (in-plane) [e]<br>10.7 ± 0.80 (in-plane) [f]<br>38.3 ± 0.83 (cross-plane) [f]	0.38 – 0.15

[a] Various vibration modes, including in-plane and out-of-plane. χ are negative for all materials here. [b] All forms of strong anharmonic behaviour suppressed. [c] Measured by X-ray diffraction. Rubrene not included. [d] See **Figure S22**, **Table S1** for details. [e] Evaluated by Raman spectroscopy with Grüneisen parameter found to be 0.87 for $N = 2$. [f] Measured by X-ray diffraction (**Figure 4c,d, Section 3.1**).

4. Computation

4.1. Lattice contribution to thermal conductivity

In **Equation (1) of the main manuscript**, the total linewidths have several contributions: $\Gamma(\mathbf{q})_s = \Gamma^{ep}(\mathbf{q})_s + \Gamma^{anh}(\mathbf{q})_s + \Gamma^{imp}(\mathbf{q})_s + \Gamma^{L}(\mathbf{q})_s$, which represent electron-phonon scattering[17,18,33–41] ($\Gamma^{ep}(\mathbf{q})_s$), third-order anharmonic phonon-phonon scattering[19,20,22,42–46] ($\Gamma^{anh}(\mathbf{q})_s$), isotopic impurity disorder[21] ($\Gamma^{imp}(\mathbf{q})_s$), and phonon-disorder scattering[22,47–49] having characteristic lengthscale L, $\Gamma_s^L(\mathbf{q}) = \frac{||\mathbf{v}(\mathbf{q})_{s,s}||}{L}$, where $||\mathbf{v}(\mathbf{q})_{s,s}||$ is modulus of the diagonal elements of the velocity operator (the group velocity of phonon with wavevector $\mathbf{q}$ and mode s). The anharmonic linewidth[50] is given by:

$$\hbar\Gamma^{anh}(\mathbf{q})_s = \frac{\pi}{N_c^2}\sum_{\mathbf{q}',\mathbf{q}'',s',s''}\{2[\bar{N}(\mathbf{q}')_{s'} - \bar{N}(\mathbf{q}'')_{s''}]\delta[\omega(\mathbf{q})_s + \omega(\mathbf{q}')_{s'} - \omega(\mathbf{q}'')_{s''}] + [1 + \bar{N}(\mathbf{q}')_{s'} + \bar{N}(\mathbf{q}'')_{s''}]\,\delta[\omega(\mathbf{q})_s - \omega(\mathbf{q}')_{s'} - \omega(\mathbf{q}'')_{s''}]\} \times$$

$$\left|\sum_{\alpha,\alpha',\alpha'',b,b',b'',\mathbf{R}',\mathbf{R}''}\frac{\partial^3 V}{\partial u_{\mathbf{0}b\alpha}\partial u_{\mathbf{R}'b'\alpha'}\partial u_{\mathbf{R}''b''\alpha''}}\mathcal{E}(\mathbf{q})_{s,b\alpha}\mathcal{E}(\mathbf{q}')_{s',b'\alpha'}\mathcal{E}(\mathbf{q}'')_{s'',b''\alpha''} \times \sqrt{\frac{\hbar^3}{8}}\frac{\Delta(\mathbf{q}+\mathbf{q}'+\mathbf{q}'')e^{-i[\mathbf{q}\cdot\boldsymbol{\tau}_b+\mathbf{q}'\cdot(\mathbf{R}'+\boldsymbol{\tau}_{b'})+\mathbf{q}''\cdot(\mathbf{R}''+\boldsymbol{\tau}_{b''})]}}{\sqrt{M_b M_{b'} M_{b''}\omega(\mathbf{q})_s\omega(\mathbf{q}')_{s'}\omega(\mathbf{q}'')_{s''}}}\right|^2$$

where $\bar{N}(\mathbf{q})_s = \left[\exp\left(\frac{\hbar\omega(\mathbf{q})_s}{k_B T}\right) - 1\right]^{-1}$ is the Bose-Einstein distribution, δ is the Dirac delta, $\frac{\partial^3 V}{\partial u_{\mathbf{0}b\alpha}\partial u_{\mathbf{R}'b'\alpha'}\partial u_{\mathbf{R}''b''\alpha''}}$ are the third-order atomic force constants, $\mathcal{E}(\mathbf{q})_{s,b\alpha}$ is the value of eigenvector of vibration $\mathbf{q}, s$ for an atom with index b and in direction α, $\Delta(\mathbf{q}+\mathbf{q}'+\mathbf{q}'')$ is a Kronecker delta equal to 1 if $\mathbf{q}+\mathbf{q}'+\mathbf{q}''$ is a reciprocal lattice vector and 0 otherwise. $M_b = \sum_i f_{i,b} m_{i,b}$ is the average atomic mass, where $f_{i,b}$ and $m_{i,b}$ are the fraction and mass of the ith isotope of atom b.

The linewidth due to isotopic impurities[50] is given by:

$$\hbar\Gamma^{imp}(\mathbf{q})_s = \frac{\hbar\pi}{2N_c}[\omega(\mathbf{q})_s]^2\sum_{\mathbf{q}',s'}\delta[\omega(\mathbf{q})_s - \omega(\mathbf{q}')_{s'}] \times \sum_b g_2^b\left|\sum_\alpha \mathcal{E}^*(\mathbf{q})_{s,b\alpha}\,\mathcal{E}(\mathbf{q}')_{s',b\alpha}\right|^2$$

where $g_2^b = \sum_i f_{i,b}\left(\frac{M_b - m_{i,b}}{M_b}\right)^2$ is a normalised mass variance of the isotopic masses of atom b. Finally, the electron-phonon linewidth[51] depends on the following quantities:

$$\hbar\Gamma^{ep}(\mathbf{q})_s = \frac{4\pi}{N_\mathbf{k}}\sum_{\mathbf{k}mn}|g_{mns}(\mathbf{k},\mathbf{q})|^2\left(f_{n\mathbf{k}} - f_{m\mathbf{k}+\mathbf{q}}\right)\delta\left(\epsilon_{m\mathbf{k}+\mathbf{q}} - \epsilon_{n\mathbf{k}} - \hbar\omega(\mathbf{q})_s\right)$$

where $N_\mathbf{k}$ is the number of electronic $\mathbf{k}$-points over which sum $\sum_{\mathbf{k}mn}$ is taken, m and n are the electronic modes, $g_{mns}(\mathbf{k},\mathbf{q})$ is the electron-phonon coupling matrix[51], $f_{m\mathbf{k}} = \left[\exp\left(\frac{\epsilon_{m\mathbf{k}} - \mu}{k_B T}\right) + 1\right]^{-1}$ is the Fermi-Dirac distribution, $\epsilon_{m\mathbf{k}}$ is electron's energy in state $m, \mathbf{k}$ and μ is the chemical potential.

In **Figure S23**, we show the phonon dispersion and magnitude of the linewidths for model of Cu-BHT with 60 atoms. We note that the phonon dispersion is positive across both in-plane and out-of-plane directions, showcasing that the structure is stable. In **Figure S23c**, we show that both the phonon-phonon and electron-phonon linewidths increase with temperature and that phonon-phonon linewidth due to third-order anharmonicity and isotopic impurities dominates over the electron-phonon contribution. This results in the bulk conductivity being weakly affected by inclusion of the electron-phonon coupling as shown in **Figure S24**.

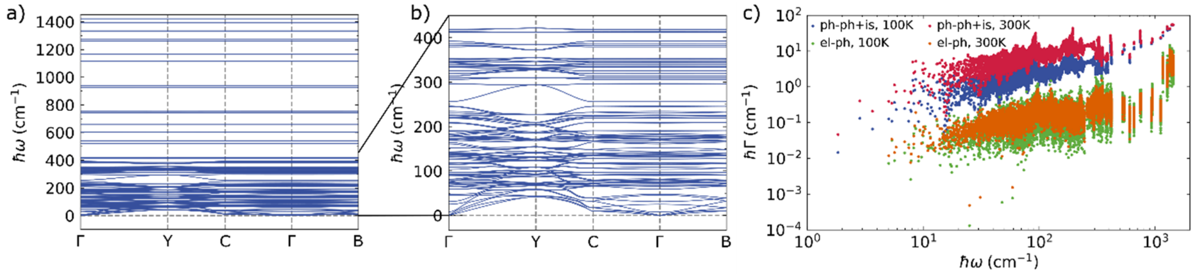

Figure S23. Phonon dispersion and magnitude of phonon linewidths in Cu-BHT. **a**, Phonon dispersion across the entire frequency range. **b**, Zoomed-in phonon dispersion in the frequency range most important for thermal transport around and below room temperature (0 – 450 cm^{-1}). **c**, Magnitude of phonon linewidths. phonon linewidths from phonon-phonon third-order anharmonic interactions and mass isotope disorder (ph-ph + is) and from electron-phonon interactions (el-ph) at temperatures of 100 and 300K. We highlight how electron-phonon linewidths are much smaller (practically negligible) compared to the phonon-phonon (ph-ph + is) linewidths.

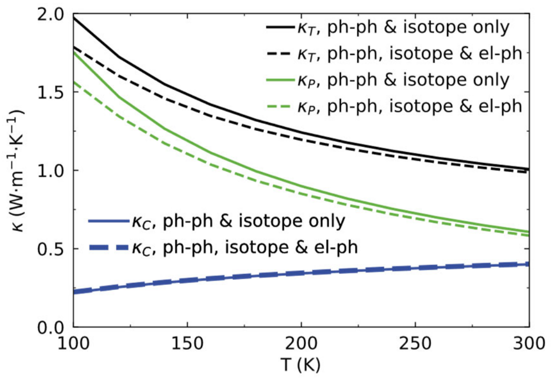

Figure S24. Electron-phonon scattering negligibly affects the bulk lattice thermal conductivity of Cu-BHT. Solid lines are lattice thermal conductivity predictions performed accounting only for phonon-phonon scattering mechanisms (third-order anharmonicity and isotopic impurities). Dashed lines are lattice thermal conductivity predictions accounting for both phonon-phonon and electron-phonon scattering. κ_P and κ_C denote the populations (particle-like propagation) and coherences (wave-like tunneling) contributions to the total conductivity, $\kappa_T = \kappa_P + \kappa_C$.

5. Thermoelectric characterizations and analysis

5.1. Thermoelectric analysis by regular and molecular Wiedemann-Franz laws

The plot of total thermal conductivities κ versus electrical conductivities σ of different compositions shows a good linear relationship, as shown in **Figure S25**. Following **Equation (S17)**

$$\kappa = \kappa_{\mathrm{ele}} + \kappa_{\mathrm{latt}} = \sigma L T + \kappa_{\mathrm{latt}} \quad \text{(S17)}$$

which states total thermal conductivity κ is a sum of electronic thermal conductivity κ_{ele} and lattice thermal conductivity κ_{latt}, and the electronic contribution can be further expanded by the Wiedemann-Franz (W-F) law $\kappa/\sigma = LT$. The Lorenz number L of our Cu-BHT films is extracted to be $(2.77 \pm 0.27)\times 10^{-8}$ V^2 K^{-2}, perfectly matched with the theoretical value of 2.44×10^{-8} V^2 K^{-2} for metals at room temperature, suggesting the electronic conduction in this material is similar to free electron motion in metals. Given that Cu-BHT is a polymeric system, we also evaluate it by using a derived W-F law for molecular system [52] where electronic conduction is dominated not by free electron but instead by inelastic electron transfer (hopping) between molecular sites. This molecular W-F law is given by **Equation (S18)** [52]

$$\kappa/\sigma = (k_{\mathrm{B}}/e)^2\, \lambda/k_{\mathrm{B}} = L_{\mathrm{M}} T_{\mathrm{M}} \quad \text{(S18)}$$

where L_{M} is the Lorenz number for molecules, T_{M} is the effective temperature, and λ is the reorganization energy, an important physical parameter relating the electron-phonon coupling in electron transfer processes. λ is found to be 0.32 meV only, corresponding to $T_{\mathrm{M}} = 3.73$ K, implying a negligible electron-phonon interaction. However, it is questionable whether this form of the W-F law for molecular systems is applicable to Cu-BHT and we use **Equation (S17)** in the following. These findings are compatible with theoretical calculations for lattice conductivity, where we found that electron-phonon contribution to phonon linewidth is much weaker compared to phonon-phonon one.

Since we find the Wiedemann-Franz law is valid in this material system, i.e. the extracted Lorenz number of $(2.77 \pm 0.27)\times 10^{-8}$ V^2 K^{-2} perfectly matches with the theoretical value of 2.44×10^{-8} V^2 K^{-2} for metals at room temperature, the electronic and lattice contributions to the total thermal conductivities are evaluated from the Wiedemann-Franz law.

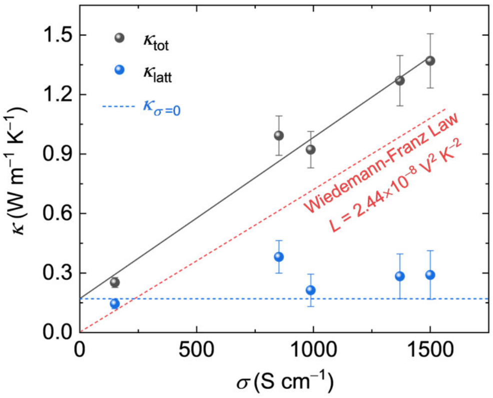

Figure S25. Plot of total thermal conductivities against electrical conductivities of Cu-BHT films. It shows a good linear relationship with a fitted $L = (2.77 \pm 0.27) \times 10^{-8}$ V^2 K^{-2}. The red, dashed line represents the W-F law with $L = 2.44 \times 10^{-8}$ V^2 K^{-2} for metal, by which the electronic and the lattice contributions are estimated. The blue short dashed line represent our experimental thermal conductivity at insulating state (zero electrical conductivity, i.e. static lattice contribution). The error bars for total thermal conductivity reflect 7% uncertainty associated with the repeatability of the measurements mainly due to the variability in thermal contacts and the measurement accuracy of the equipment. Combined with the ~7% uncertainty in the measurement accuracy for the determination of the electronic contribution, the error bars for the lattice thermal conductivity are obtained in quadrature.

5.2. Summary of electrical conductivity, lattice thermal conductivity, and thermoelectric performance

Table S3. Summary of electrical conductivity σ and lattice thermal conductivity κ_{latt} of different types of thermoelectric materials at near room temperature. This table does not include materials in which thermoelectric enhancement is attributed to using special technologies such as nanostructuring and aligning.

Materials	κ_{latt} (W m^{-1} K^{-1})	σ (S cm^{-1})	Reference
Organic materials			
PEDOT-Tos	0.31	85	[53]
P3HT	0.31	45	[54]
PDPPSe-12	0.27	252	[55]
PBTTT	0.41	520	[55]
PBTTT	0.50	700	[56]
TBDOPV-T	0.36	85	[57]
Inorganic materials			
Bi_2Te_3 alloy	0.79	833	[58]
Sb_2Te_3 alloy	0.66	869	[58]
Mg_3Bi_2 alloy	0.59	556	[58]
Mg_3Bi_2 alloy	1.8	1234	[59]
Mg_3Bi_2 alloy	1.4	1000	[59]
Mg_3Bi_2 alloy	0.9	625	[59]
Mg_3Bi_2 alloy	0.8	487	[59]
Mg_3Bi_2 alloy	0.7	409	[59]
Commercial Bi_2Te_3 alloy	0.88	1111	[59]
Commercial Bi_2Te_3 alloy	0.76	740	[59]
SnSe crystal	0.69	11	[60]
Cage-like compounds such as skutterudites			
$LaFe_3CoSb_{12}$	0.76	666	[61]
$CeFe_3CoSb_{12}$	0.56	666	[61]
$Ce_{0.17}Co_4Sb_{12}$	2.4	2170	[62]
$Ce_{0.18}Co_4Sb_{12}$	2.2	2170	[62]
$Ce_{0.20}Co_4Sb_{12}$	2.2	2000	[62]
$Ce_{0.21}Co_4Sb_{12}$	2.1	1515	[62]
$Co_4Sb_{11.46}Te_{0.43}$	3.9	1700	[63]
$S_{0.26}Co_4Sb_{11.11}Te_{0.73}$	1.5	1100	[63]
$Se_{0.17-m}Co_4Sb_{11.31}Te_{0.53}Se_m$	2.5	1070	[63]
$Br_{0.16}Co_4Sb_{11.34}Te_{0.52}$	3.4	1350	[63]
$S_{0.18}Co_{3.4}Ni_{0.58}Sb_{11.94}$	2.2	1020	[63]
Open frameworks such as porous MOFs			
Ni-HATP	0.35	92	[64]
Ni-HATP	0.21	60	[65]
TCNQ@$Cu_3(BTC)_2$	0.28	0.0043	[66]
Non-open, non-porous Cu-BHT films			
Cu-BHT films (Cu/BHT=2) *	0.38	636	(this work)
Cu-BHT films (Cu/BHT=3) *	0.29	1534	(this work)
Cu-BHT films (Cu/BHT=4.5) *	0.28	1459	(this work)
Cu-BHT films (Cu/BHT=5.5) *	0.21	1500	(this work)
Cu-BHT films (Cu/BHT=7) *	0.14	150	(this work)
Cu-BHT films (previous work)	0.15	147	[67]

* The molar ratio of Cu^{2+}/BHT added into the reaction (not found value)

Table S4. Summary of electrical conductivity and Seebeck coefficient of different Cu/BHT ratios.

Cu/BHT ratio	***σ*** **(S cm^{-1})**	***S*** **(μV K^{-1})**	Cu/BHT ratio	***σ*** **(S cm^{-1})**	***S*** **(μV K^{-1})**
2	414	7.29	4	1520	3.5
	435	8.27		2240	2.6
	417	8.96		2272	2.65
	836	6.1	4.5	1390	1.42
	807	4.96		1760	1.11
3	1200	5.43		1990	2.47
	1960	4.36		1340	2.72
	1100	5.56		1090	2.28
	1170	5.42	5	1490	3.17
	1200	6.3		2050	1.4
	1960	6.29		1640	1.88
	2070	4.22	5.5	843	4.62
	2250	3.49		2320	1.87
	2230	3.14		843	4.1
3.5	2280	2.73		1346	4.24
	2167	2.78	6	856	4.6
	2216	3.42		1240	3.37
	2220	2.92		1530	2.47
	1910	1.94	6.5	462	6.26
	1960	3.23		567	7.22
	1940	2.6	7	358	10.2
				615	7.05

5.3. Carrier transport analysis with mobility edge, variable-range hopping, heterogeneous transport, and semi-localized transport models

We have tried to use mobility edge (ME) model [68,69] to understand and quantify the weakly thermally activated transport behavior. This model, expressed as **Equation (S19)**,

$$\sigma = \sigma_{\mathrm{min}} \exp\left(-\frac{E_\mathrm{a}}{k_\mathrm{B}T}\right) \tag{S19}$$

gives an activation energy E_a varying from 2.0 meV at room temperature, 0.01 meV at 10 K, and even to zero below 10 K (**Figure S26a**); this phenomenon has been observed in previous studies of Cu-BHT [11] and of another one-dimensional coordination polymer NiTTFtt [70] more recently as well. However, such a large deviation from linear relationship between semi-logarithmic plot of σ against $1/T$ and the non-constant E_a cannot be understood by classical ME model that predicts linear relationship and constant activation energy [68,69].

We also looked into applying variable-range hopping (VRH) models [71,72] which are commonly used for charge transport in disordered semiconductors and amorphous solid and have been applied to interpret the temperature dependence of transport coefficients of coordination polymer NiTTFtt [70] and organic polymers such as PBTTT [73]. We find that, different-dimension VRH models (d = 2, 3) given by **Equation S20** as well as the Efros and Shklovskii (ES) model (d = 1) [71,72] given by **Equation S21** cannot give good fit for the whole temperature regime (**Figure S27a – c**).

$$\sigma = \sigma_0 \exp\left(-\frac{T_\mathrm{M}}{T}\right)^{1/(d+1)},\ d = 2, 3 \tag{S20}$$

$$\sigma = \sigma_0 \exp\left(-\frac{T_\mathrm{ES}}{T}\right)^{1/(d+1)},\ d = 1 \tag{S21}$$

To further analyse this Zabrodskii analysis[74] was also performed. If the conductivity could be well described by **Equations (S20), (S21)**, the logarithmic derivative should take the form of **Equations (S22), (S23)** with $a = 1/(d+1)$:

$$\log W \approx \log(aT_0^a) - a\log T \tag{S22}$$

$$W = \frac{d\log\sigma}{d\log T} \tag{S23}$$

However, the value of a extracted from the Zabrodskii analysis is too small to have a physical dimension meaning and becomes negative at about < 220 K (**Figure S27d,e**), which is usually a sign of the presence of metallic states. This result is reproducible in 2 individual devices fabricated with 2 different batches of synthesis. This result, meanwhile, mathematically, means there is an unknown large term in T^b with $b > 0$ in place, especially at low temperature, in addition to the hopping-related thermal activation. This lead us turn back to the ME model, since Mott had proposed that in degenerate systems if electron-electron interaction play a role (especially at low temperature), there is a need to introduce a correction term $\Delta\sigma \propto T^{1/2}$ into the ME model [68,69]. The long-range electron-electron Coulomb interaction may modify the density of states, we here, however, introduce the correction term into the electrical conductivity in the simplest manner, as **Equation (S24)** [68,69] where A is a fitting parameter, to better understand the temperature dependence of the conductivity behavior in our material system.

$$\sigma = \sigma_{\mathrm{min}} \exp\left(-\frac{E_\mathrm{a}}{k_\mathrm{B}T}\right) + AT^{1/2} \tag{S24}$$

Surprisingly, it provides a very good fit for the weak, positive temperature dependence of conductivity (**Figure S26b**), with σ_{min} indeed being smaller than the fit result without the correction term, as predicted by Mott. A clearer, more careful justification for taking the contribution of electron-electron interaction will be further explored and discussed in heterogeneous transport model [75].

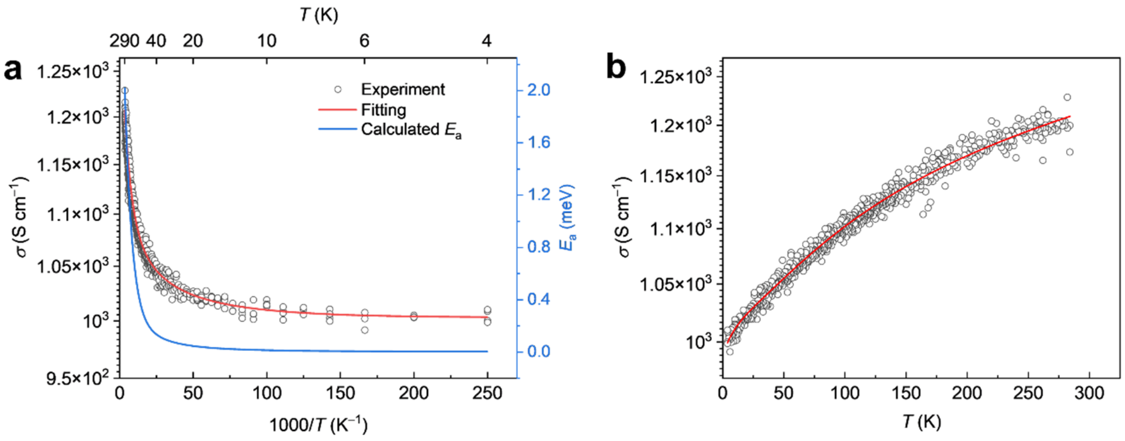

Figure S26. Conductivity analysis for Cu/BHT mole ratio 2 with mobility edge model. **a**, Fit by classical mobility edge model by **Equation (S19)**. **b**, Fit with the correction term of electron-electron interaction by **Equation (S24)**.

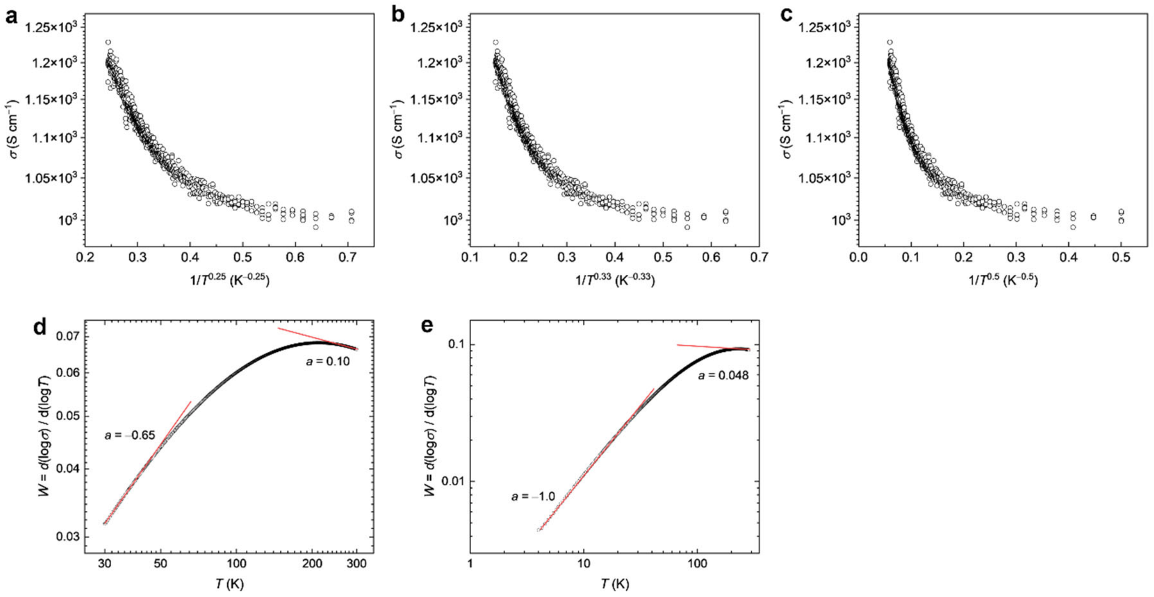

Figure S27. Conductivity analysis for Cu/BHT mole ratio 2 with VRH model. **a – c**, Plots with transport dimensions $d = 3$, 2, and 1 respectively. **d** and **e**, More systematical Zabrodskii analysis performed on two individual samples by **Equations (S22), (S23)**. They both exhibit $a < 0$ at about < 220 K, suggesting the presence of metallic carriers.

In heterogeneous transport model [75], conduction can be written as the sum of the resistances of the metallic parts (first term, subscript m) of ordered, crystalline regions and the barrier parts (second term) of disordered regions. Considering the fact that metallic-thermally activated transition may occur in the barrier parts, with hopping (subscript h) and disordered metallic (subscript d) transport present in parallel, this model gives the electrical conductivity as

$$\sigma^{-1} = (g_{\mathrm{m}}\sigma_{\mathrm{m}})^{-1} + (g_{\mathrm{h}}\sigma_{\mathrm{h}} + g_{\mathrm{d}}\sigma_{\mathrm{d}})^{-1} \tag{S25}$$

where $g_{\mathrm{i}} = \frac{LA_{\mathrm{i}}}{L_{\mathrm{i}}A}$, is a geometry factor. L_{i} and A_{i} are the effective length and cross section area of the conducting path. The full expression is

$$\sigma(T)^{-1} = (g_{\mathrm{m}}\sigma_{\mathrm{m0}})^{-1}\exp\left(-\frac{T_{\mathrm{m}}}{T}\right) + \left[g_{\mathrm{h}}\sigma_{\mathrm{h0}}\exp\left(-\frac{T_0}{T}\right)^{\gamma} + \left(g_{\mathrm{d}}\sigma_{\mathrm{d}} + g_{\mathrm{d}}\alpha T^{\frac{1}{2}}\right)\right]^{-1} \tag{S26}$$

where σ_{i0} is conductivity prefactor, T_m is the energy of phonons that scatter carriers, T_0 and γ are the hopping energy and dimension in variable-range hopping model respectively, and $g_d \alpha T^{\frac{1}{2}}$ is for the potentially present electron-electron (e-e) interaction term that belongs to the disordered metallic transport regions. As shown in **Figure S28**, we find that as Cu/BHT ratio increases, the metallic contribution increases, consistent with the temperature-dependent phenomena among different compositions. This result indicates that the transport in the crystalline samples is more strongly grain boundary limited, while in the amorphous samples the gran boundaries dominate the transport less.

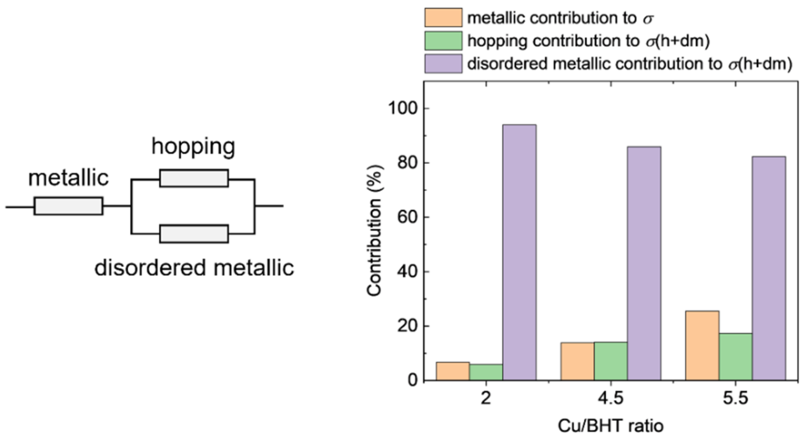

Figure S28. Transport analysis with heterogeneous transport model on electrical conductivity of Cu-BHT films synthesized with different Cu/BHT ratio. Electron-electron interaction is considered here. The actual fit for the more strongly grain boundary limited, crystalline sample can be found in **Figure 3h,i**. The contributions of hopping transport (green) and disordered metallic transport (purple) represent their relative contributions to charge transport at grain boundaries only (i.e. hopping contribution + disordered metallic contribution = 100%, happening at grain boundaries). The contribution of metallic transport (orange) happening within grains represents the contribution to total charge transport. The bar chart represents the fitting result of the single measurements of different Cu/BHT ratios.

Semi-localized transport (SLoT) model was recently developed by Gregory *et al.* [76] to interpret the temperature-dependent transport coefficient and the relation between electrical conductivity σ and Seebeck coefficient S in organic conducting polymers. This model was developed based on the Boltzmann transport formalism and captures the energy-dependent behaviour by assuming $\sigma_E(E,T,c)$ to take the following form:

$$\sigma = \int_{-\infty}^{\infty} \sigma_E(E,T)\left(-\frac{df}{dE}\right) dE \tag{S27}$$

$$S = \frac{1}{\sigma}\left(\frac{k_B}{e}\right) \int_{-\infty}^{\infty} \sigma_E(E,T)\left(\frac{E-E_F}{k_B T}\right)\left(-\frac{df}{dE}\right) dE \tag{S28}$$

$$\sigma_E(E,T,c) = \begin{cases} 0, (E < E_t) \\ \sigma_0 \exp\left(-\frac{W_H(c)}{k_B T}\right) \times \left(\frac{E-E_t}{k_B T}\right)^s, (E \geq E_t) \end{cases} \tag{S29}$$

where f is the Fermi-Dirac distribution function, E_F is the Fermi energy level, e is the fundamental charge, and k_B is the Boltzmann constant. $\sigma_E(E,T)$ is the transport function describing electrical conduction at energy E and temperature T and, importantly, relating the two important transport

coefficients, i.e. electrical conductivity and Seebeck coefficient. $W_H(c)$ is the localization energy, i.e. the depth of the potential well for charge transport.

We attempt to use this model to fit the weakly temperature-activated electrical conductivity in Cu-BHT films. We evaluate the relative position of the Fermi level with respect to the transport level by using the measured Seebeck coefficient of ~8 μV/K at room temperature and assuming $s = 1$ that has been used for highly conducting organic conjugated polymers. The result with an extracted value of 34 for a reduced Fermi level $|E - E_t|/(kT)$ is shown in **Figure S29**. Apparently, the SLoT model fails with Cu-BHT films. It is most likely due to the assumption of a single (parabolic) band that has been used in the SLoT model, which is not applicable to Cu-BHT films because charge transport in Cu-BHT films is in a multi-band, hole-electron competing transport regime (will discuss in **Section 6** in details).

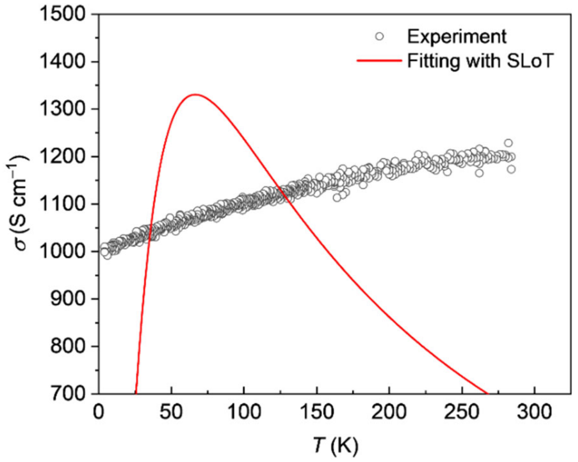

Figure S29. Conductivity analysis for Cu/BHT mole ratio 2 with SLoT model. The fit is done with an extracted value of 34 for a reduced Fermi level $|E - E_t|/(kT)$ according to the measured Seebeck coefficient of ca. 8 μV/K at room temperature [76]. Apparently this model is not applicable to Cu-BHT films.

5.4. Carrier transport analysis with Boltzmann transport equation

In this work, we use Boltzmann transport equation with Fermi-Dirac distribution function including scattering mechanisms based on below considerations:

1. We do believe our system is degenerate and metallic, as supported by the non-zero conductivity at near 0 K, extremely small Seebeck coefficient, comparable Lorenz number to metals', negative correlation between electrical conductivity and temperature, and clear magnetoresistance and Hall effect.
2. Existence of energy-dependent transport events, as seen in the thermally activated conductivity and super-linear Seebeck coefficient as a function of temperature, indicating the presence of thermally activated events within band transport.

For the scattering mechanisms, we have considered electron-electron interaction, trapped carrier/ionic impurity scattering, neutral defect scattering, boundary scattering, optical phonon scattering, acoustic phonon scattering, and scatterings by optical and acoustic phonons-induced potential deformations. They have different scattering parameter s in a simple energy dependent scattering of the form

$$\tau(\epsilon) = \tau_s(\epsilon/k_B T)^s \quad \text{(S30)}$$

where ϵ is the energy level and τ is the energy-dependent scattering time. Among above scattering mechanisms, (optical and acoustic) phonons scatterings and scatterings by phonons-induced potential deformations are negatively correlated with charge transport over temperature [77,78] ($s < 0$); they therefore most likely govern the charge transport in the disordered, amorphous compositions where negatively correlated electrical conductivity and temperature is seen. Neutral defect scattering is usually mild at low temperature and represented by a constant relaxation time [77,78] ($s = 0$), which is inconsistent with our observation that shows stronger scattering at low temperature for crystalline compositions. Hence, electron-electron interaction, trapped charge scattering, and boundary scattering are most likely. Since the presence of long-range electron-electron interaction is usually determined by carrier density rather than chemical and structural orderings, we don't expect this scattering only appears in our crystalline sample. Similarly, it is difficult to understand trapped charge scattering only dominates the charge transport in the crystalline, ordered sample but not in the amorphous, disordered one. Combined with the fact that the most crystalline composition has the most serious grain boundary issue, as evident by their morphologies shown in **Figure 2c,d**, it is reasonable to believe that scattering at grain boundaries (with or without trapped charges at which) is the major mechanism.

To deal with this, there are two ways:

1. Energy filtering, where extremely strong scattering is viewed as an energy filtering effect in charge transport. By this low-energy carriers can be preferentially blocked by an energy barrier, i.e. simply remove their contributions to thermoelectric transport [78,79].
2. Band electrons experiencing scattering in extended states, leading to only a fraction of carriers participates in charge transport, since charge carriers can be significantly localized due to strong scattering [77].

We cannot exclude the potentially possible influence of the changes in density of states, however we emphasize the boundary scattering should play a role.

5.5. Influences of impurities in BHT on thermoelectric properties

The impurities in the starting material BHT have non-negligible influences on the final thermoelectric properties of the films. BHT, that appears light yellow indicating the presence of impurities, provides higher Seebeck coefficients and lower electrical conductivities than white BHT at the same Cu/BHT mole ratio loading (**Figure S30**). The peak values are about twice in Seebeck coefficients and half in electrical conductivities, with respect to those without impurities. As seen in **Figure S31**, apparently, the impurities impede the carrier transport reaching the metallic region and give rise to a stronger thermal activation transport behavior. We note that sometime the impurities can be colorless, so that the color of the BHT is not an indicator for purity and the final performance of the films. Careful chemical and/or molecular structure characterizations are needed in this case.

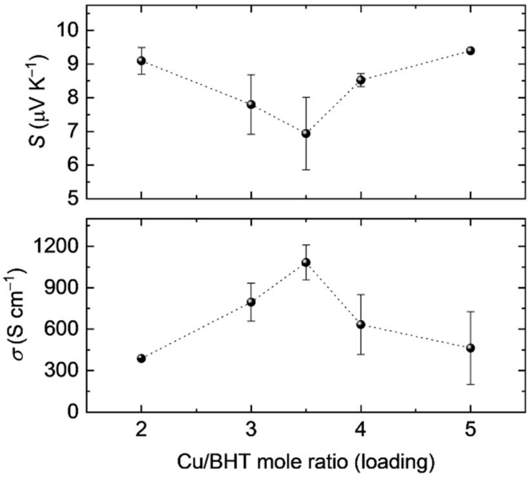

Figure S30. Seebeck coefficients and electrical conductivities as a function of Cu/BHT mole ratio of the films synthesized with light yellow BHT. Data are presented as mean values and the error bars present the standard deviation of samples. These values are higher in Seebeck coefficient and lower in electrical conductivity than those of the films synthesized with white BHT which can be found in **Figure 2**.

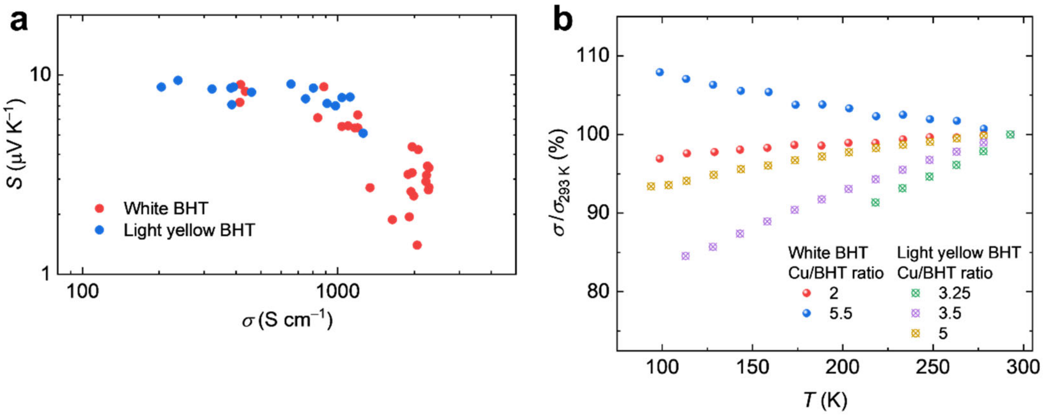

Figure S31. Comparison of the films synthesized with white and with light yellow BHT in thermoelectric properties. **a**, Plot of Seebeck coefficient versus electrical conductivity. The data presented here is for Cu/BHT ratio 2 – 5. **b**, Normalized temperature-dependent electrical conductivities.

6. Magnetotransport characterization and analysis

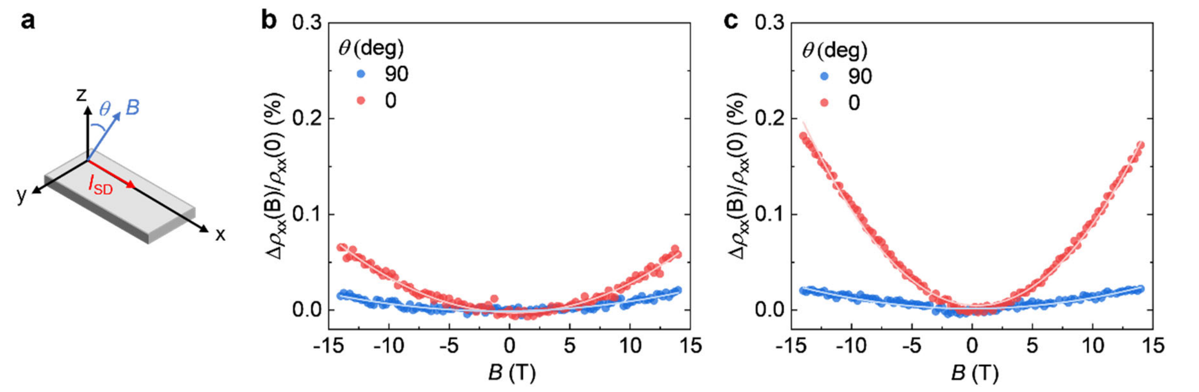

Figure S32. Angular-dependent magnetoresistance of Cu-BHT films. **a**, Measurement architecture, where $\theta = 0$ and 90 deg represent a field perpendicular and parallel to the current respectively. **b** and **c**, Plots of MR against B for crystalline Cu/BHT ratio 2 and amorphous Cu/BHT ratio 5 respectively.

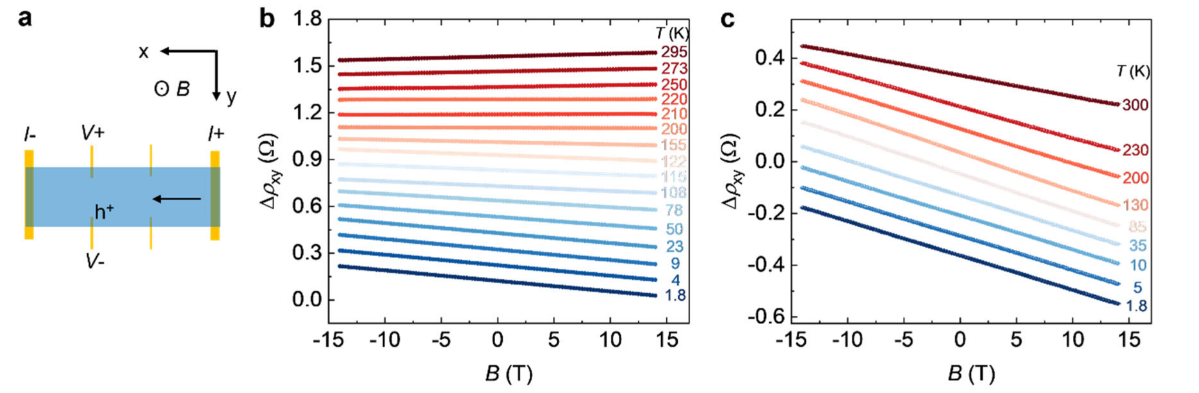

Figure S33. Temperature-dependent DC Hall effect of Cu-BHT films. **a**, Hall bar device architecture and the electrical connection used in the measurements. The magnetic field was always perpendicular to the films. **b** and **c**, Hall resistance as a function of magnetic field and temperature for crystalline samples (Cu/BHT ratio of 2) and amorphous samples (Cu/BHT ratio of 5), respectively.

The Hall coefficient of the amorphous (Cu/BHT ratio 5) is positive and exhibits a peak around 150K, while the Hall coefficient of the crystalline (Cu/BHT ratio 2) sample changes sign around 210 K from an electron-dominated, high temperature regime to a hole-dominated, low temperature one (**Figure 5d**). These phenomena suggest that near the peak of the amorphous sample charge transport is dominated by hole carriers, but as temperature increases the Hall coefficient drops as electrons start to compete [80,81]. This is with the demonstrated moving up of the Fermi level from amorphous to crystalline samples (**Figure S16**), a hole-electron competing transport regime is likely to be applicable in the crystalline composition.

To understand the influences of the defect-modified electronic structure on the nature of the carriers, we have developed a two-carrier transport framework to interpret the Hall and magnetoresistance transport data in Cu-BHT. Such a two-carrier analysis is an underdetermined problem and it is not possible to unambiguously determine the carrier concentrations n_e and n_h and the corresponding mobilities μ_e and μ_h, respectively without making some simplifying assumptions:

1. If the low-temperature regime of the amorphous sample is in the single carrier governed regime, the hole carrier concentration can be easily extracted to be 2.8 per unit cell by $R_H = 1/ne$; this

result means in two-carrier transport regime the hole concentration cannot be larger than this value since at this point the density of states for hole is the highest, according to the metallic double-band electronic structure (**Figure 5e**). In our modeling, hence, we use 2.8 per unit cell as the upper bound.

2. Kohler's plot is satisfied either in a single carrier model with a single scattering time or in a two-carrier model where two carriers' transport is resonant and their temperature dependences are almost the same over a wide range of temperature [82,83]. In both cases, the Kohler's plots at different temperature should gather, otherwise the curves separate from each other [82,84]. Our plots are mostly apart for both crystalline and amorphous samples. Combined with the Hall coefficient analysis, it can be concluded that it is quite possible electron-hole competing transport takes place in both samples. To simply the scenario, herein, we assume in two-carrier regime $b\mu_e(T) = \mu_h(T)$ (represents Hall mobility) and b is an adjustable scaling factor.
3. Mobilities evaluated from magnetoresistance and from Hall effect are usually different. They are linked by magnetoresistance scattering factor $\Upsilon = \mu_{MR}/\mu_H$, with Υ always >1 and usually ranging from 1 – 2. Its value depends on scattering mechanism [85]. In our simulation we therefore manage to get $\Upsilon \sim 1 - 2$ fitted, or relax to 3 at maximum if necessary.
4. Several observations already indicate electronic conduction in this material system for both crystalline and amorphous compositions are delocalized charge carriers experiencing different-level scatterings in diffusive, metallic regime. We therefore give the temperature-dependent mobility a functional form of $\mu = AT^x$, with $-1.5 < x < 0$. The upper bound of 0 ensure it is metallic transport while the lower bound of –1.5 represents the perfect extreme of acoustic phonon scattering only (for optical phonon scattering $x = -0.5$) [77,78]. A value between –1.5 and 0 means there is other thermally activated term(s) in play, such as boundary and charge impurity scatterings, consistent with our experimental observations.

In two-carrier transport at weak field limit (strong scattering regime) where $\omega_c\tau \ll 1$ $\left(\mu_{e,h}^{-1} \gg B\right)$ [86] with $b\mu_e = \mu_h$, electrical conductivity, Hall coefficient, and magnetoresistance are given by [83,84,87,88]

$$\sigma_{xx}(B) = \sigma_{n,xx}(B) + \sigma_{p,xx}(B) = \frac{e}{B}\left[\frac{n_e\omega_{c,e}\tau_e}{1+(\omega_{c,e}\tau_e)^2} + \frac{n_h\omega_{c,h}\tau_h}{1+(\omega_{c,h}\tau_h)^2}\right] = \frac{en_e\mu_e}{1+\mu_e^2B^2} + \frac{en_h\mu_h}{1+\mu_h^2B^2} = \frac{en_e\mu_e}{1+\mu_e^2B^2} + \frac{ebn_h\mu_e}{1+b^2\mu_e^2B^2} \quad \text{(S31)}$$

$$R_H = -\frac{1}{e}\frac{n_e\mu_e^2 - n_h\mu_h^2}{(n_e\mu_e + n_h\mu_h)^2} = -\frac{1}{e}\frac{n_e - b^2n_h}{(n_e + bn_h)^2} \quad \text{(S32)}$$

$$\frac{\rho_{xx}-\rho_0}{\rho_0} = \frac{n_e\mu_e n_h\mu_h}{(n_e\mu_e + n_h\mu_h)^2}(\mu_e + \mu_h)^2B^2 = \frac{bn_en_h}{(n_e+bn_h)^2}(b+1)^2{\mu_e}^2B^2 \quad \text{(S33)}$$

where n_e and n_h, μ_e and μ_h, τ_e and τ_h, and $\omega_{c,e}$ and $\omega_{c,h}$ are carrier concentrations, mobilities, scattering times, and cyclotron frequencies for electron and hole respectively, and e is the elementary charge.

Based on this basic hypothesis we developed a two-carrier transport model that is consistent with our experimental observations and allows us to estimate hole and electron carrier concentrations and mobilities:

1. Plot the magnetoresistance scattering factor Υ as a function of A at different possible b values (**Figure S34a-c**), from which the upper and lower bounds of A at different b that allows Υ falling within a scientifically reasonable range of 1 – 3 can be extracted.
2. Plot the hole density using the extracted A and b. Since the upper bound estimated from the single carrier regime suggests the hole concentration cannot be larger than 1.45×10^{21} cm^{-3}, this can further confirm the minimum (maximum) possible value for b (A).
3. Since the A and b values we screened out should work at different temperatures, we can then get a plot of A against b directly showing the possible values (**Figure S34g**).

4. Since a computed band structure of Cu-BHT reported previously shows a more dispersive electron band than hole bands, b should < 1, i.e. higher electron mobilities than hole mobilities.
5. Plot the simulated electron and hole mobilities form Hall effect using the bounds, as displayed in the main text.

Similar simulation was also performed on the crystalline Cu/BHT ratio of 2. Although the simulated result is a little diverging, however, the tendency is clear and consistent that electron has higher mobility and lower concentration than hole. The resulting n_e, n_h, μ_e, μ_h are summarized in **Figure 5**.

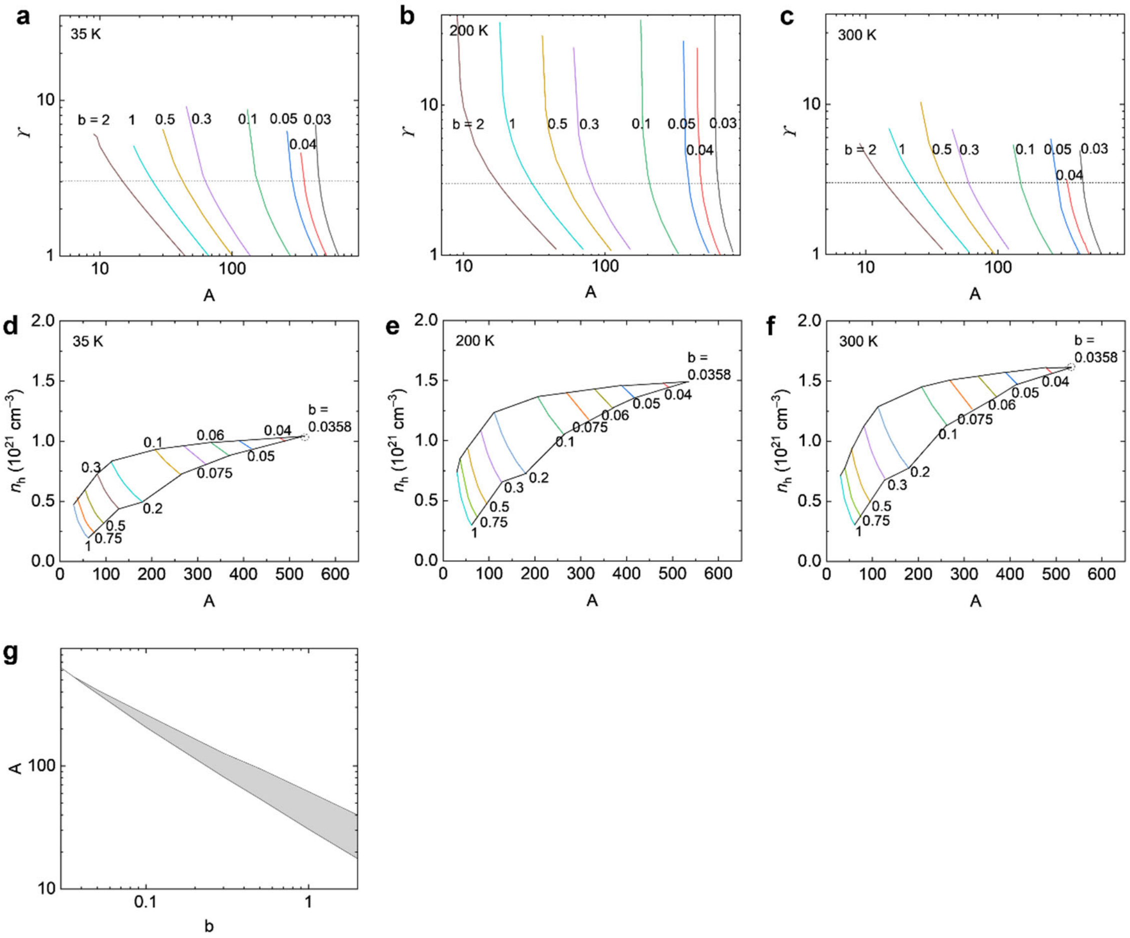

Figure S34. Simulation of Hall mobilities and carrier concentrations of the amorphous Cu/BHT ratio of 5. a – c, Plot of magnetoresistance scattering factor Υ against A at different b at 35, 200, and 300 K. **d – f**, Hole concentration as a function of A at different b at 35, 200, and 300 K. The black box presents the possible range. **g**, Polt of A against b extracted from Figures **a – c** showing the possible range of A and b values within the bound of Υ< 3.

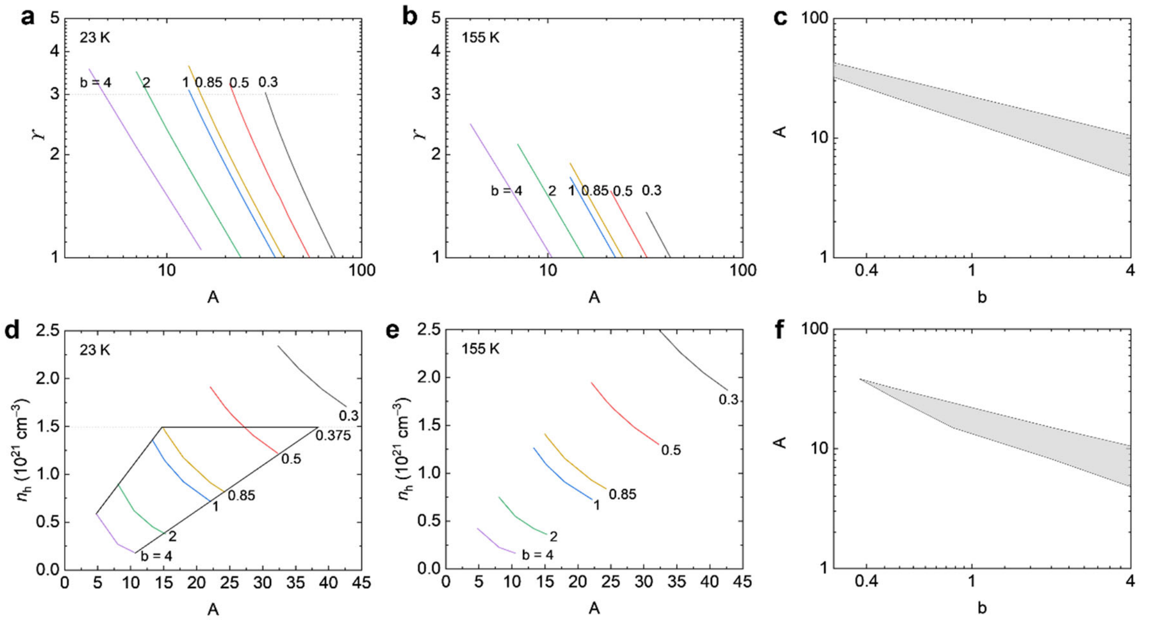

Figure S35. Simulation of Hall mobilities and carrier concentrations of the crystalline Cu/BHT ratio of 2. a, b, Plot of magnetoresistance scattering factor Υ against A at different b at 23 and 155 K. **c**, Polt of A against b extracted from Figures **a, b** showing the reasonable range of A and b values within the bound of $\Upsilon < 3$. **d, e**, Hole concentration as a function of A at different b at 200 and 300 K. The black box presents the reasonable range. **f**, Further narrowed A and b values after considering the bound of hole concentration.

Supplementary References

1. Toyoda, R. *et al.* Heterometallic Benzenehexathiolato Coordination Nanosheets: Periodic Structure Improves Crystallinity and Electrical Conductivity. *Adv. Mater.* **34**, 2106204 (2022).
2. Pan, Z. *et al.* Synthesis and structure of a non-van-der-Waals two-dimensional coordination polymer with superconductivity. *Nat. Commun.* **15**, 9342 (2024).
3. Huang, X. *et al.* Superconductivity in a Copper(II)-Based Coordination Polymer with Perfect Kagome Structure. *Angew. Chemie* **130**, 152–156 (2018).
4. Jiang, Z. GIXSGUI: A MATLAB toolbox for grazing-incidence X-ray scattering data visualization and reduction, and indexing of buried three-dimensional periodic nanostructured films. *J. Appl. Crystallogr.* **48**, 917–926 (2015).
5. Ferrer Orri, J. *et al.* Unveiling the Interaction Mechanisms of Electron and X-ray Radiation with Halide Perovskite Semiconductors using Scanning Nanoprobe Diffraction. *Adv. Mater.* **34**, 2200383 (2022).
6. D. N. Johnstone, P. Crout, S. Høgås, B. Martineau, J. Laulainen, S. Smeets, S. Collins, E. Jacobsen, J. Morzy, E. Prestat, T. Doherty, T. Ostasevicius, H. W. Ånes, T. Bergh, R. Tovey, E. O. pyxem/pyxem: pyxem 0.12.0. https://doi.org/10.5281/zenodo.3968871.
7. Ophus, C. *et al.* Automated Crystal Orientation Mapping in py4DSTEM using Sparse Correlation Matching. *Microsc. Microanal.* **28**, 390–403 (2022).
8. Venkateshvaran, D. *et al.* Approaching disorder-free transport in high-mobility conjugated polymers. *Nature* **515**, 384–388 (2014).
9. Statz, M. *et al.* On the manifestation of electron-electron interactions in the thermoelectric response of semicrystalline conjugated polymers with low energetic disorder. *Commun. Phys.* **1**, 16 (2018).
10. Linseis, V., Völklein, F., Reith, H., Nielsch, K. & Woias, P. Advanced platform for the in-plane ZT measurement of thin films. *Rev. Sci. Instrum.* **89**, 015110 (2018).
11. Huang, X. *et al.* A two-dimensional π-d conjugated coordination polymer with extremely high electrical conductivity and ambipolar transport behaviour. *Nat. Commun.* **6**, 7408 (2015).
12. Paolo, G. *et al.* QUANTUM ESPRESSO: a modular and open-source software project for quantum simulations of materials. *J. Phys. Condens. Matter* **21**, 395502 (2009).
13. Giannozzi, P. *et al.* Advanced capabilities for materials modelling with Quantum ESPRESSO. *J. Phys. Condens. Matter* **29**, 465901 (2017).
14. Prandini, G., Marrazzo, A., Castelli, I. E., Mounet, N. & Marzari, N. Precision and efficiency in solid-state pseudopotential calculations. *npj Comput. Mater.* **4**, 72 (2018).
15. Pickard, C. J. & Needs, R. J. Ab initio random structure searching. *Journal of Physics Condensed Matter* 053201 at https://doi.org/10.1088/0953-8984/23/5/053201 (2011).
16. Eriksson, F., Fransson, E. & Erhart, P. The Hiphive Package for the Extraction of High-Order Force Constants by Machine Learning. *Adv. Theory Simulations* **2**, 1800184 (2019).
17. Lee, H. *et al.* Electron–phonon physics from first principles using the EPW code. *npj Comput. Mater.* **9**, 156 (2023).
18. Cepellotti, A., Coulter, J., Johansson, A., Fedorova, N. S. & Kozinsky, B. Phoebe: a high-performance framework for solving phonon and electron Boltzmann transport equations. *JPhys Mater.* **5**, 035003 (2022).
19. Carrete, J. *et al.* almaBTE : A solver of the space–time dependent Boltzmann transport equation for phonons in structured materials. *Comput. Phys. Commun.* **220**, 351–362 (2017).
20. Togo, A. & Seko, A. On-the-fly training of polynomial machine learning potentials in computing lattice thermal conductivity. *J. Chem. Phys.* **160**, 211001 (2024).
21. Tamura, S. I. Isotope scattering of dispersive phonons in Ge. *Phys. Rev. B* **27**, 858 (1983).
22. Fugallo, G., Lazzeri, M., Paulatto, L. & Mauri, F. Ab initio variational approach for evaluating lattice thermal conductivity. *Phys. Rev. B - Condens. Matter Mater. Phys.* **88**, 045430 (2013).
23. Pizzi, G. *et al.* Wannier90 as a community code: New features and applications. *J. Phys. Condens. Matter* **32**, 165902 (2020).
24. Marzari, N., Mostofi, A. A., Yates, J. R., Souza, I. & Vanderbilt, D. Maximally localized Wannier functions: Theory and applications. *Rev. Mod. Phys.* **84**, 1419 (2012).
25. Atomic-Precision Non-van der Waals 2D Structures: Superconductivity in. (2024).

26. Rivnay, J., Noriega, R., Kline, R. J., Salleo, A. & Toney, M. F. Quantitative analysis of lattice disorder and crystallite size in organic semiconductor thin films. *Phys. Rev. B - Condens. Matter Mater. Phys.* **84**, 1–20 (2011).
27. Prosa, T. J., Moulton, J., Heeger, A. J. & Winokur, M. J. Diffraction line-shape analysis of poly(3-dodecylthiophene): a study of layer disorder through the liquid crystalline polymer transition. *Macromolecules* **32**, 4000–4009 (1999).
28. Warren, B. E. & Averbach, B. L. The separation of cold-work distortion and particle size broadening in x-ray patterns [1]. *J. Appl. Phys.* **23**, 497 (1952).
29. Zobeiri, H. *et al.* Effect of temperature on Raman intensity of nm-thick WS2: Combined effects of resonance Raman, optical properties, and interface optical interference. *Nanoscale* **12**, 6064–6078 (2020).
30. Asher, M. *et al.* Chemical Modifications Suppress Anharmonic Effects in the Lattice Dynamics of Organic Semiconductors. *ACS Mater. Au* **2**, 699–708 (2022).
31. Huang, X. *et al.* Quantitative Analysis of Temperature Dependence of Raman shift of monolayer WS2. *Sci. Rep.* **6**, 1–8 (2016).
32. Sahoo, S., Gaur, A. P. S., Ahmadi, M., Guinel, M. J. F. & Katiyar, R. S. Temperature-dependent Raman studies and thermal conductivity of few-layer MoS2. *J. Phys. Chem. C* **117**, 9042–9047 (2013).
33. Kumar, S., Multunas, C., Defay, B., Gall, D. & Sundararaman, R. Ultralow electron-surface scattering in nanoscale metals leveraging Fermi-surface anisotropy. *Phys. Rev. Mater.* (2022) doi:10.1103/PhysRevMaterials.6.085002.
34. Maliyov, I., Yin, J., Yao, J., Yang, C. & Bernardi, M. Dynamic mode decomposition of nonequilibrium electron-phonon dynamics: accelerating the first-principles real-time Boltzmann equation. *npj Comput. Mater.* **10**, 1–8 (2024).
35. Protik, N. H., Li, C., Pruneda, M., Broido, D. & Ordejón, P. The elphbolt ab initio solver for the coupled electron-phonon Boltzmann transport equations. *npj Comput. Mater.* (2022) doi:10.1038/s41524-022-00710-0.
36. Giustino, F., Cohen, M. L. & Louie, S. G. Electron-phonon interaction using Wannier functions. *Phys. Rev. B - Condens. Matter Mater. Phys.* (2007) doi:10.1103/PhysRevB.76.165108.
37. Poncé, S., Jena, D. & Giustino, F. Hole mobility of strained GaN from first principles. *Phys. Rev. B* (2019) doi:10.1103/PhysRevB.100.085204.
38. Noffsinger, J. *et al.* EPW: A program for calculating the electron-phonon coupling using maximally localized Wannier functions. *Comput. Phys. Commun.* (2010) doi:10.1016/j.cpc.2010.08.027.
39. Poncï¿½, S., Margine, E. R., Verdi, C. & Giustino, F. EPW: Electron–phonon coupling, transport and superconducting properties using maximally localized Wannier functions. *Comput. Phys. Commun.* (2016) doi:10.1016/j.cpc.2016.07.028.
40. Sundararaman, R. *et al.* JDFTx: Software for joint density-functional theory. *SoftwareX* (2017) doi:10.1016/j.softx.2017.10.006.
41. Verdi, C. & Giustino, F. Fröhlich electron-phonon vertex from first principles. *Phys. Rev. Lett.* (2015) doi:10.1103/PhysRevLett.115.176401.
42. Paulatto, L., Mauri, F. & Lazzeri, M. Anharmonic properties from a generalized third-order ab initio approach: Theory and applications to graphite and graphene. *Phys. Rev. B - Condens. Matter Mater. Phys.* (2013) doi:10.1103/PhysRevB.87.214303.
43. Tadano, T., Gohda, Y. & Tsuneyuki, S. Anharmonic force constants extracted from first-principles molecular dynamics: Applications to heat transfer simulations. *J. Phys. Condens. Matter* (2014) doi:10.1088/0953-8984/26/22/225402.
44. Li, W., Carrete, J., Katcho, N. A. & Mingo, N. ShengBTE: A solver of the Boltzmann transport equation for phonons. *Comput. Phys. Commun.* (2014) doi:10.1016/j.cpc.2014.02.015.
45. Jain, A. Multichannel thermal transport in crystalline Tl3VSe4. *Phys. Rev. B* (2020) doi:10.1103/PhysRevB.102.201201.
46. Xia, Y. *et al.* High-Throughput Study of Lattice Thermal Conductivity in Binary Rocksalt and Zinc Blende Compounds including Higher-Order Anharmonicity. *Phys. Rev. X* (2020)

doi:10.1103/PhysRevX.10.041029.
47. Fugallo, G. *et al.* Thermal conductivity of graphene and graphite: Collective excitations and mean free paths. *Nano Lett.* (2014) doi:10.1021/nl502059f.
48. Casimir H.B.G. Note on the conduction on heat in crystals. *Physica* (1938).
49. Born, M., Huang, K. & Lax, M. Dynamical Theory of Crystal Lattices. *Am. J. Phys.* (1955) doi:10.1119/1.1934059.
50. Pazhedath, A., Bastonero, L., Marzari, N. & Simoncelli, M. First-principles characterization of thermal conductivity in LaPO4-based alloys. *Phys. Rev. Appl.* **10**, 1 (2023).
51. Giustino, F. Electron-phonon interactions from first principles. *Rev. Mod. Phys.* (2017) doi:10.1103/RevModPhys.89.015003.
52. Craven, G. T. & Nitzan, A. Wiedemann-Franz Law for Molecular Hopping Transport. *Nano Lett.* **20**, 989–993 (2020).
53. Bubnova, O. *et al.* Optimization of the thermoelectric figure of merit in the conducting polymer poly(3,4-ethylenedioxythiophene). *Nat. Mater.* **10**, 429–433 (2011).
54. Kroon, R. *et al.* Bulk Doping of Millimeter-Thick Conjugated Polymer Foams for Plastic Thermoelectrics. *Adv. Funct. Mater.* **27**, 1704183 (2017).
55. Wang, D. *et al.* Multi-heterojunctioned plastics with high thermoelectric figure of merit. *Nature* **632**, 528–535 (2024).
56. Zhu, W. *et al.* Enhancing the Conductivity and Thermoelectric Performance of Semicrystalline Conducting Polymers through Controlled Tie Chain Incorporation. *Adv. Mater.* **36**, 2310480 (2024).
57. Lu, Y. *et al.* Persistent Conjugated Backbone and Disordered Lamellar Packing Impart Polymers with Efficient n-Doping and High Conductivities. *Adv. Mater.* **33**, 2005946 (2021).
58. Yang, J. *et al.* Next-generation thermoelectric cooling modules based on high-performance Mg3(Bi,Sb)2 material. *Joule* **6**, 193–204 (2022).
59. Mao, J. *et al.* High thermoelectric cooling performance of n-type Mg3Bi2-based materials. *Science (80-.).* **365**, 495–498 (2019).
60. Zhao, L. D. *et al.* Ultralow thermal conductivity and high thermoelectric figure of merit in SnSe crystals. *Nature* **508**, 373–377 (2014).
61. Sales, B. C., Mandrus, D. & Williams, R. K. Filled skutterudite antimonides: A new class of thermoelectric materials. *Science (80-.).* **272**, 1325 (1996).
62. Tang, Y., Hanus, R., Chen, S. W. & Snyder, G. J. Solubility design leading to high figure of merit in low-cost Ce-CoSb3 skutterudites. *Nat. Commun.* **6**, 7584 (2015).
63. Duan, B. *et al.* Electronegative guests in CoSb3. *Energy Environ. Sci.* **9**, 2090 (2016).
64. Un, H. I. *et al.* Controlling Film Formation and Host-Guest Interactions to Enhance the Thermoelectric Properties of Nickel-Nitrogen-Based 2D Conjugated Coordination Polymers. *Adv. Mater.* **36**, 2312325 (2024).
65. Sun, L. *et al.* A Microporous and Naturally Nanostructured Thermoelectric Metal-Organic Framework with Ultralow Thermal Conductivity. *Joule* **1**, 168–177 (2017).
66. Erickson, K. J. *et al.* Thin film thermoelectric metal-organic framework with high seebeck coefficient and low thermal conductivity. *Adv. Mater.* **27**, 3453–3459 (2015).
67. Tsuchikawa, R. *et al.* Unique Thermoelectric Properties Induced by Intrinsic Nanostructuring in a Polycrystalline Thin-Film Two-Dimensional Metal–Organic Framework, Copper Benzenehexathiol. *Phys. Status Solidi Appl. Mater. Sci.* **217**, 2000437 (2020).
68. Mott, N. F. The conductivity near a mobility edge. *Philos. Mag. B Phys. Condens. Matter; Stat. Mech. Electron. Opt. Magn. Prop.* **49**, 75–82 (1984).
69. Mott, S. N. The mobility edge since 1967. *J. Phys. C Solid State Phys. 20* **20**, 3075–3102 (1987).
70. Xie, J. *et al.* Intrinsic glassy-metallic transport in an amorphous coordination polymer. *Nature* **611**, 479–484 (2022).
71. Hopping, V. Variable-Range Hopping. **601**, 601–613 (1976).
72. Van Lien, N. & Dinh Toi, D. *Coulomb correlation effects in variable-range hopping thermopower*. *Physics Letters A* vol. 261 www.elsevier.nlrlocaterphysleta (1999).
73. Ito, H., Mada, H., Watanabe, K., Tanaka, H. & Takenobu, T. Charge transport and thermoelectric conversion in solution-processed semicrystalline polymer films under

electrochemical doping. *Commun. Phys.* **4**, 8 (2021).
74. Zabrodskii, A. G. & Zinov'eva, K. N. Low-Temperature Conductivity and Metal-Insulator Transition in Compensate n-Ge. *Sov. Phys. JETP* **59**, 425–433 (1984).
75. Kaiser, A. B. Systematic conductivity behavior in conducting polymers: Effects of heterogeneous disorder. *Adv. Mater.* **13**, 927–941 (2001).
76. Gregory, S. A. *et al.* Quantifying charge carrier localization in chemically doped semiconducting polymers. *Nat. Mater.* **20**, 1414–1421 (2021).
77. Wang, X., Register, L. F. & Dodabalapur, A. Redefining the Mobility Edge in Thin-Film Transistors. *Phys. Rev. Appl.* **11**, 1–13 (2019).
78. Berland, K. *et al.* Enhancement of thermoelectric properties by energy filtering: Theoretical potential and experimental reality in nanostructured ZnSb. *J. Appl. Phys.* **119**, 125103 (2016).
79. Tran, V. A., Tran, P. A., Nguyen, H. Q., Bach, G. H. & Nguyen, T. T. Boundary-scattering induced Seebeck coefficient enhancement in thin films within relaxation time approximation. *Phys. B Condens. Matter* **635**, 413800 (2022).
80. LeBoeuf, D. *et al.* Electron pockets in the Fermi surface of hole-doped high-Tc superconductors. *Nature* **450**, 533–536 (2007).
81. Androulakis, J. *et al.* Thermoelectric enhancement in PbTe with K or Na codoping from tuning the interaction of the light- and heavy-hole valence bands. *Phys. Rev. B - Condens. Matter Mater. Phys.* **82**, 1–8 (2010).
82. Ishiwata, S. *et al.* Extremely high electron mobility in a phonon-glass semimetal. *Nat. Mater.* **12**, 512–517 (2013).
83. Huang, S. M., Yu, S. H. & Chou, M. Two-carrier transport-induced extremely large magnetoresistance in high mobility Sb2Se3. *J. Appl. Phys.* **121**, 015107 (2017).
84. Li, C. Z. *et al.* Two-Carrier Transport Induced Hall Anomaly and Large Tunable Magnetoresistance in Dirac Semimetal Cd3As2 Nanoplates. *ACS Nano* **10**, 6020–6028 (2016).
85. Kyriakos, D. S., Valassiades, O. E. & Economou, N. A. Weak-field magnetoresistance coefficients and the related magnetoresistance skewness effect. *Rev. Phys. Appliquée* **17**, 49–54 (1982).
86. Akiba, K. *et al.* Two-carrier analyses of the transport properties of black phosphorus under pressure. *Phys. Rev. B* **95**, 1–7 (2017).
87. Wang, Y. *et al.* Direct Evidence for Charge Compensation-Induced Large Magnetoresistance in Thin WTe2. *Nano Lett.* **19**, 3969–3975 (2019).
88. Luo, Y. *et al.* Hall effect in the extremely large magnetoresistance semimetal WTe2. *Appl. Phys. Lett.* **107**, (2015).
